# Multitemporal modeling and simulation of the complex dynamics in urban wetlands: the case of Bogota, Colombia

**DOI:** 10.1038/s41598-023-36600-8

**Published:** 2023-06-09

**Authors:** Yenny Cuellar, Liliana Perez

**Affiliations:** Laboratoire de Géosimulation Environnementale (LEDGE), Département de Géographie, Université de Montréal, 1375 Avenue Thérèse-Lavoie-Roux, Montréal, QC H2V 0B3 Canada

**Keywords:** Environmental sciences, Environmental impact

## Abstract

Urban wetlands are essential to the longstanding health and well-being of cities. Acknowledged as rich in biodiversity and highly productive ecosystems, they provide ecosystem services represented in aspects such as air purification, urban climate regulation, physical and mental health, recreation, and contemplation, among a wide variety of other goods and services on which the quality of life of the inhabitants of large cities such as Bogota depends largely. We used cellular automata to model and simulate urban wetland changes in Bogota, Colombia. The study applied the coupled Markov-Future Land Use Simulation (FLUS) model to simulate and analyze land use/land cover (LULC) change over 20 years. First, we used an orthomosaic (1998) and two WorldView-2 satellite images (2004 and 2010), to detect land cover changes. Then, using the artificial neural network FLUS module, we calculated the relationships between land classes and associated drivers and estimated the probability of occurrence of each land class. Finally, we applied Intensity Analysis to examine the observed and projected LULC change (1998–2034). Results indicate that gains in areas of crops and pastures are at the expense of wetlands. In addition, simulation outputs show that wetlands will likely represent less than 2% of the total study area in 2034, representing a 14% decrease in 24 years. The importance of this project lies in its potential contribution to the decision-making process within the city and as an instrument of natural resource management. Additionally, the results of this study could contribute to the United Nations Sustainable Development Goal 6, “Clean water and sanitation," and climate change mitigation.

## Introduction

Founded in and around cities or their suburbs^1^, urban wetlands are unique and play an indispensable role in the well-being of the urban area. They offer multiple ecosystem services such as water supply, water purification, diminishing the urban heat island effect, providing habitat to critical plant and animal species, flood regulation, and recreational opportunities^2,3^. However, those ecosystems face threats, such as sewage pollution, changed water regimes, reduced hydrological functions, habitat and biodiversity loss or climate change, and loss due to land-use change^4^. Moreover, in previous decades, individuals and societies have disregarded the value of wetlands^5^, filling them with municipal and construction waste material and seeing them as a source of insecurity. For example, in Bogota, the area of wetlands in the 1940s was 50 000 hectares, but today it stands at only 901 hectares^6–8^.

In 1950, 70% of the world's population lived in rural areas, but it was until 2007 that the urban population surpassed this number^9^. Ever since, the urban population has continued growing, and United Nations estimates it will increase to 68% by 2050^9^. Urban growth is increasing environmental pressure, especially in and around urban centers^10^. Latin America and the Caribbean are amongst the most urbanized regions in the world, where nearly 81% of the inhabitants live in cities^11^. In Colombia, during the 60 s and 70 s of the twentieth century, urban growth was fueled mainly by migration from the countryside to the city due to violence^12^. Since the late 1950s, the city of Bogota has experienced substantial growth and spread westward, reaching today the Bogota River. As development and urban growth increase, protected wetlands—that once were in the peripheral or rural areas—are absorbed by the city, losing their environmental and ecological qualities^10^. Thus, preserving and protecting these ecosystems is crucial because of their inherent role in monitoring global change and guiding human adaptation to a changing world^9^.

To retain and restore urban wetlands, studies on the changes in urban wetlands have gained momentum since the 2000s, and researchers have analyzed mainly historical changes in their landscape patterns and ecological functions. Fieldwork, remotely sensed data, Geographic Information Systems (GIS), and complexity science modeling approaches have been applied to evaluate changes in these ecosystems^5,7,13–19^. The complex interactions and relationships among the environmental components that drive wetland formation and regulation require methodological approaches to detect and analyze wetland changes across spatial and temporal scales in complex urban landscapes. Land Use Land Cover (LULC) change models using Cellular Automata (CA) are increasingly used to explore land change dynamics. They are considered a basis for public policy decisions to promote protecting and manage ecosystems^20^.

Researchers have used computational models to understand better the spatial and temporal dynamics observed in urban wetlands. X. Wang et al.^21 ^used landscape indices and the Markov model to evaluate the relationship between changes in wetland landscapes and urban construction in Wuhan, China. Peng et al.^20^ developed a model of spatial allocation by pairing Random Forest (RF) regression and the Conversion of Land Use and its Effects (CLUE-s) model to simulate the spatial dynamics of the urban wetlands in the Wuhan Agglomeration. Ghosh & Das^22^ assessed the East Kolkata Wetland shift peril using RF and Support Vector Machine (SVM). Saha et al.^23^ mapped the floodplain wetlands of the Atreyee river basin of India and Bangladesh. They estimated their area up to 2039 using Artificial Neural Networks and Cellular automata (ANN-CA) techniques. To sum up, researchers have implemented spatiotemporal models to help decision-makers protect the wetland landscape.

There is a broad spectrum of studies on LULC changes in Bogota City. Cabrera-Amaya et al.^24^ did a floristic characterization of the vegetation in the Jaboque wetland and determined the changes in the vegetation cover between 2004 and 2016. Morales^23^ conducted a multi-temporal analysis in the Santa Maria del Lago wetland in 1952, 1990, and 2014. Garzón Gutiérrez^24^ assessed the environmental conditions of the Juan Amarillo wetland using and comparing multi-temporal remote sensing data. Bernal Jaramillo^25^ studied the physical degradation of the La Vaca, Techo, and El Burro wetlands to establish landscape guidelines for their management. In summary, previous studies have aimed to study Bogota's wetlands' physical, historical, and compositional changes to draw the attention of citizens and competent entities to restore, conserve and protect them. However, to our knowledge, no studies have investigated future trends of wetland changes in Bogota.

The main goal of this research is to implement a computational model to simulate and study the changes in the urban wetlands of Bogota, Colombia. To accomplish our main objective, we: 1) interpreted one analog (1998) and two numerical images digitized (2004 and 2010) to extract LULC and estimated the changes and tendencies in the wetlands during the frame time; 2) obtained the probability maps for each land class from the application of ANN to simulate wetland changes; 3) applied a hybrid land cover model, using the LULC probability maps, the Markov Chain, and the FLUS-CA model^26^; and 4) employed the Intensity Analysis framework^27,28^ to validate the model and analyze the results.

The following section presents the study area and data for implementing the CA-FLUS model. Next, we describe the methodology, beginning with the particularities of the modeling approach and continuing with the techniques used to evaluate the observed and projected LULC changes. Then, we present the results and discuss the analysis. Finally, we depicted the conclusions and implications.

## Material and methods

### Study area

**Figure 1:**
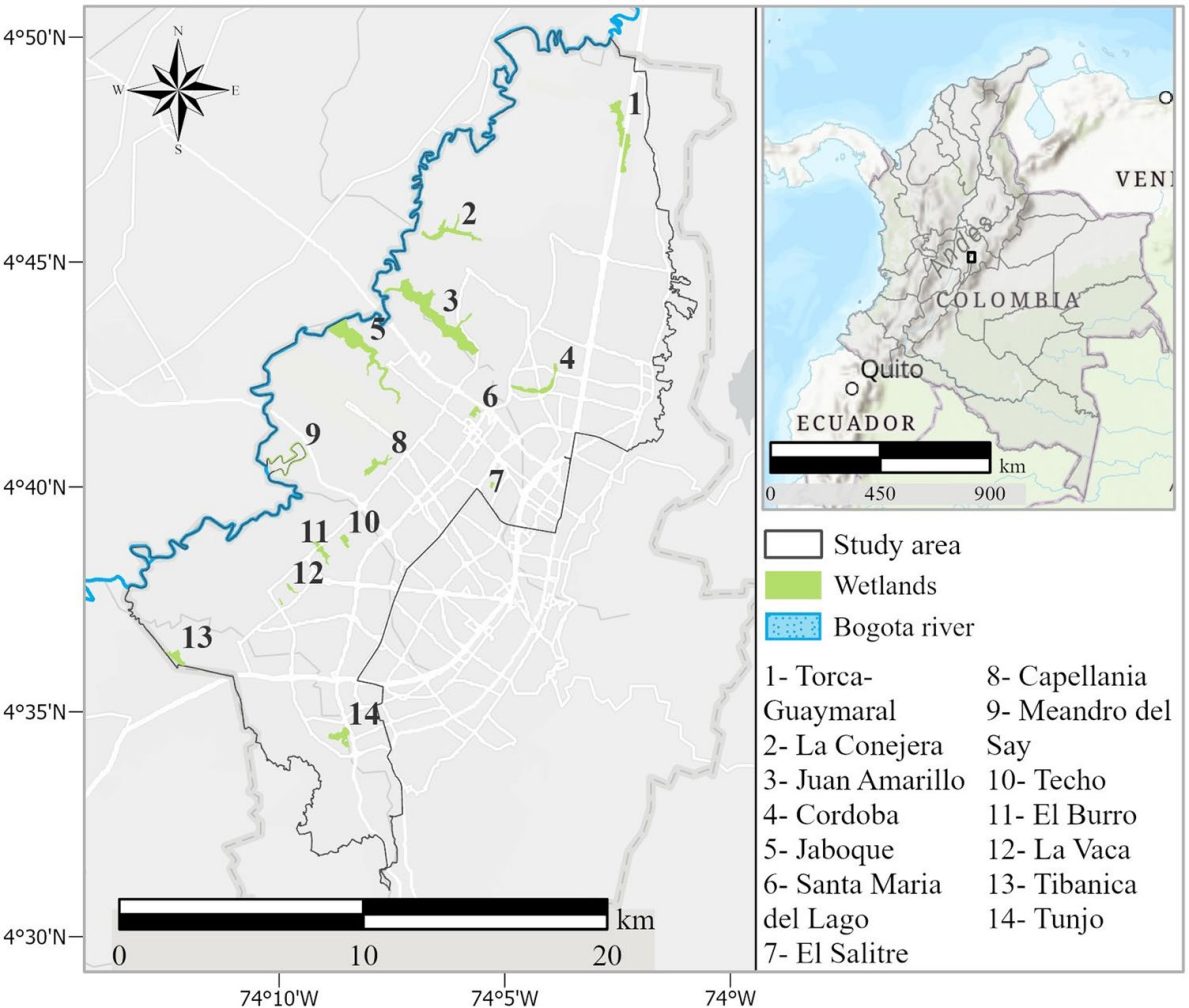
The study area of Bogota, Colombia.

Located in the Eastern Cordillera, in the northern part of the Andes, the capital city of Colombia accounts for an altitude between 2650 and 3750 masl (meters above sea level). The temperature of the city varies between 7 °C-14.5 °C. The rainfall is bimodal, with alternating periods of 2 to 3 months of rain with rainfall of 163 mm (April-June and September–November) and two dry periods with 20 mm (January–February and July–August)^29^. The urban area occupies approximately 379 km^2^ and has a particular river network. It is next to the Eastern Hills, near the paramos of Chingaza and Sumapaz. The population reaches 7,181,469^30^. The Bogota wetlands, located west of the city, are part of the historic area of ​​marshy transition with the city center. Bogota's urban wetland complex has 15 ecosystems recognized by the city's Environment Secretariat, but the Ramsar Convention recognizes only 11 with an area of ​​6.67 km^2^^31^. Figure 1 shows the location of fourteen wetlands. We took only the administrative localities that have a direct impact and are around the influenced area of the ecosystems.

### Data sources

Three images were selected from the Spatial Data Infrastructure of Bogota (IDECA), based on their temporality and resolution. The chosen remotely sensed products were: (a) for the year 1998 an orthomosaic, created from orthorectified aerial photographs, and (b) for the years 2004 and 2010 WorldView-2 satellite images. Subsequently, we performed an on-screen digitization of the images by using visual image interpretation elements and obtained the LULC maps. Then, parting from the definition of wetlands, "areas of marsh, fen, peatland or water, whether natural or artificial, permanent or temporary, with water that is static or flowing, fresh, brackish or salt, including areas of marine water the depth of which at low tide does not exceed six metres^32^″, we selected six land classes: constructions, urban green spaces, crops and pastures, quarries, water, and wetlands.

**Figure 2:**
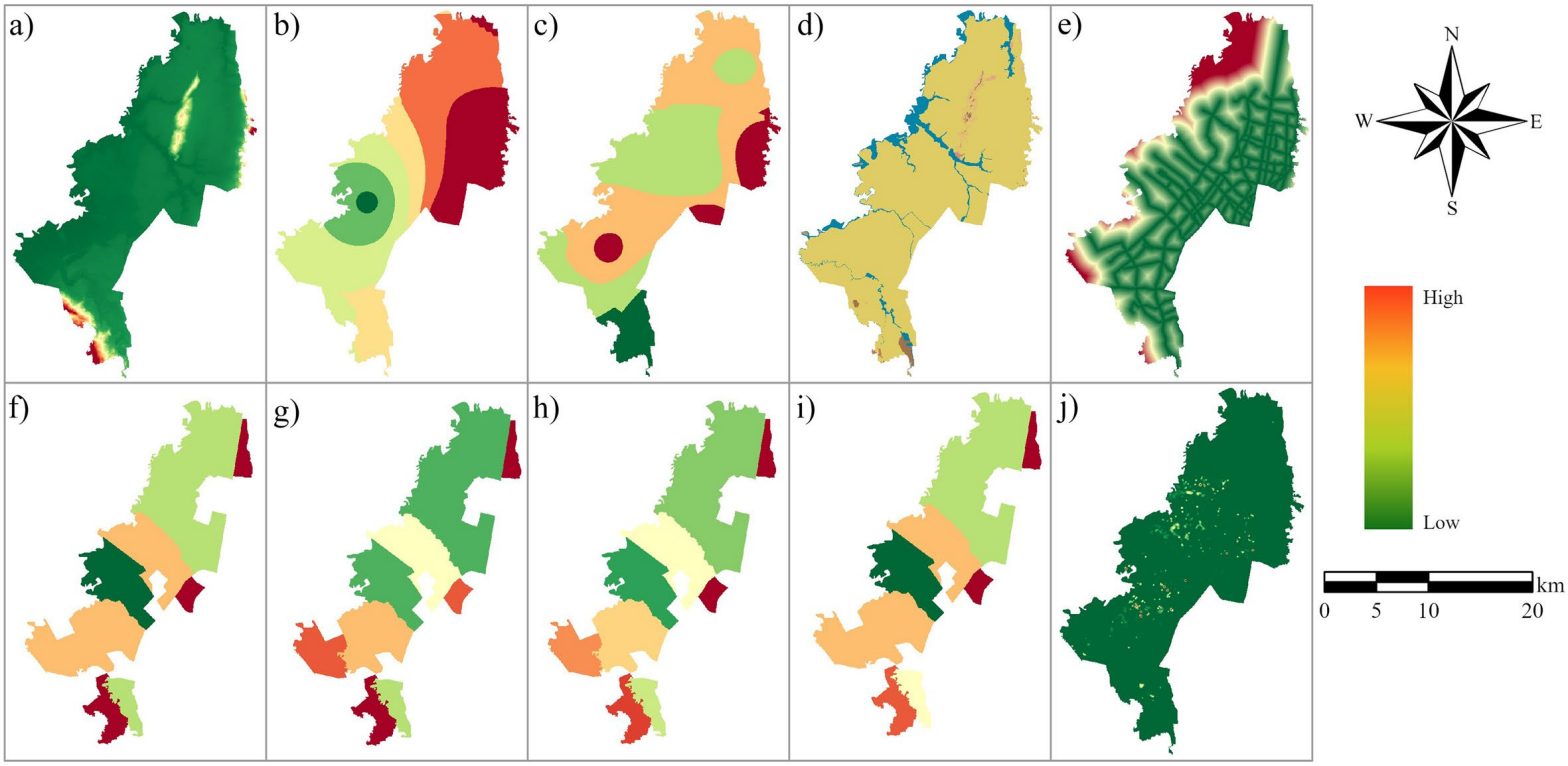
Driving factors used to simulate land use change (**a**) digital elevation model, (**b**) annual precipitation, (**c**) mean annual temperature, (**d**) urban geomorphology, (**e**) distance to roads, (**f**) population density for the year 2004, (**g**) population density for the year 2010, (**h**) household density for the year 2004, (**i**) household density for the year 2010, (**j**) cadastral information. A detailed map of urban geomorphology and the legend are attached as Supplementary Fig. S1.

Many variables affect urban wetlands^33^. Therefore, we selected the driving factors and local knowledge based on a literature review^33–36^. The driving factors identified were the distance to roads, the Digital Elevation Model (DEM), population density, household density, cadastral information, and climatic variables such as precipitation and temperature. Table 1 lists the spatial datasets used to simulate LULC in Bogota. We calculated the distance to the arterial road network using the Euclidean distance tool of ArcGIS Pro version 2.6.0^37^. The restricted area data represent the protected area system established in the city's Land Use Plan^38^. All the spatial datasets were rasterized and resampled to a five-meter pixel size. The driving factors were normalized to improve model accuracy to eliminate dimensional and quantitative differences (Fig. 2).

**Table 1:** Data resources.

Data	Year	Format	Resolution	Source and description
Orthomosaic	1998	WMS	0.5 m	Open data portal of the city of Bogota https://www.ideca.gov.co
WorldView-2 satellite imagery	2004, 2010	WMS	0.46 m	
Distance to the arterial road network		Vector	1:1000	
DEM	2014	Raster	5 m	
Urban geomorphology	2016	Vector	1:1000	
Restricted areas		Vector	1:1000	
Population	2004, 2010	xlsx	Localities*	http://www.bogota.gov.co
Cadastral information	1998, 2004, 2010	Vector	1:1000	https://serviciosgis.catastrobogota.gov.co
Annual mean temperature		Vector	1:1500	https://datosabiertos.bogota.gov.co
Annual precipitation		Vector	1:1500	

*Local administrative division of the City of Bogota.

## Methodology

We applied three methodical approaches to simulate LULC change: Markov Chain (MC), Artificial Neural Networks (ANN), and the Future Land Use Simulation (FLUS) model used successfully in the simulation of complex LULC in past research^26,39–41^.

### Land use demand projection using Markov Chain

We use the MC model as the "top-down” land-use demand forecasting module of the FLUS model^39^. The MC model is a method for projecting future land-use demands by determining the transition probability of change from one category to another in a time interval and employed in other simulation studies^17,42,43^. In this research, we implemented MC in three time periods, i.e., 2010–2016, 2016–2022, and 2022–2028.

### FLUS model

The FLUS model simulates land-use change under the effect of human existence and nature by applying a spatial simulation process based on a CA model. To set relationships between historical land-use data and the driving factors of change, it implements an ANN. The value of the probabilities of occurrence on each pixel guides the allocation of changes in land-use distribution. Then, its self-adaptive inertia and competition mechanism allow the model to develop complex local land-use interactions and competition, effectively dealing with the uncertainty and complexity of the transformation of various land-use types under different influences^26^. In addition, the FLUS model uses the Moore neighborhood to represent the neighborhood space. This study tested the model’s sensitivity using three neighborhood dimensions 3 × 3, 5 × 5, and 7 × 7. See X. Liu et al.^26^ for a detailed model description.

### Artificial neural networks

Researchers use Machine learning techniques like ANN to approximate the nonlinear and complex relationships between LULC patterns and their driving variables^42,44^. Used as data mining tools to extract land-use class transition rules for CA^45^, the ANN includes several neurons that work in parallel to transform the input data into output categories^23^—consisting of three-layer types: an input layer, a hidden layer, and an output layer. In the ANN, each neuron in the input layer conforms to the land-use maps and the driving factors. Each neuron in the output layer conforms to a distinct land type representing the probability of occurrence of each land type concerning the influencing factors. We employed the random sampling technique to extract 70% of the data as samples to ensure the same sampling points for all kinds of land. Normalized processing is carried out on the data samples and then imported into the ANN model to obtain the suitability probability of each category.

### Model implementation

**Figure 3:**
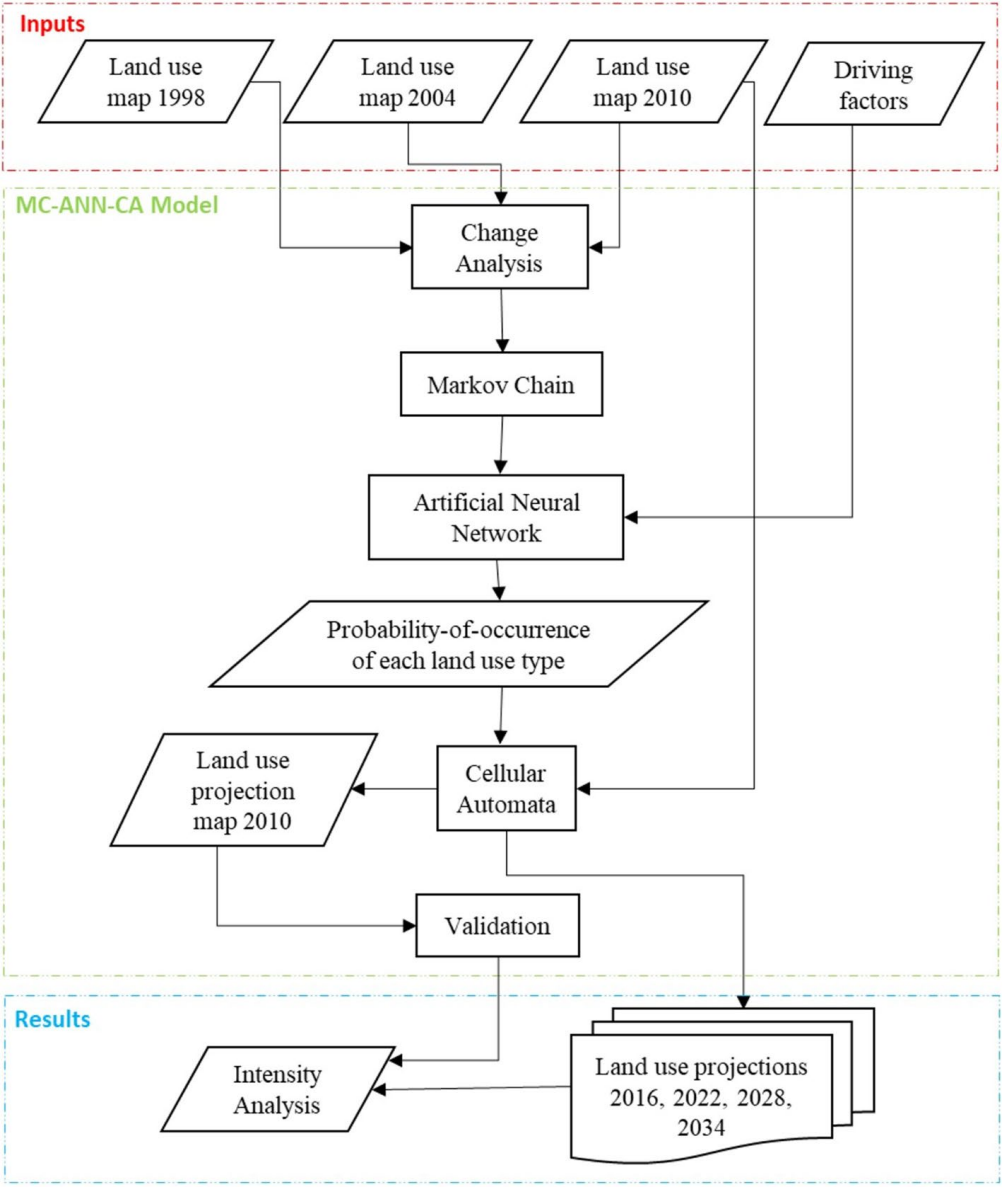
Schematic diagram of the methodology.

Figure 3 shows the steps we followed to simulate land-cover change. Firstly, we performed a change analysis to identify the most crucial land-cover class transitions in the first-time interval. We used the LULC maps from 1998 and 2004 for carrying out the change analysis.

Secondly, we utilized the MC to obtain the unknown land demand in simulated intervals. Initially, land demand was calculated based on the reference map for the year 2010. Then, subsequent time intervals were calculated based on the previous time interval. So, for example, to obtain the land demand for 2016, the transition matrix was calculated for the years 2004–2010.

Thirdly, the ANN was constructed of 10 neurons (Table 1) in the input layer (the driving factors) and six neurons in the output layer (corresponding to the land types). 70% of the total pixels across the study area were randomly selected as the training dataset^26^. Before training the network, the sampling data is normalized to [0 1]; to do it, the FLUS model uses the sigmoid function as the model transfer function to ensure the estimated probability values’ range is [0 1]^26^.

The FLUS model was run to simulate 30-year land-cover changes on the study case of urban wetland changes in Bogota, initiated in 2004. The simulation timeframe was selected to validate the results against the available LULC map corresponding to 2010. A total of five simulated maps were calculated, and an emphasis of our analysis assessment was centered around the urban wetlands category.

The following stage used the CA module to carry out LULC map projections. A 3 × 3, 5 × 5, and 7 × 7 Moore neighborhoods were used for the simulation. The CA module was implemented in two phases: calibration/validation and simulation. We calibrated and validated the model from 1998 to 2010 and performed the simulation from 2016 to 2034. First, we calibrated the model using the observed maps of 1998 and 2004. Then, to obtain the projection for the year 2010, we used the observed 2004 LULC map. Next, we validated the model by comparing the reference 2010 land-cover map with the 2010 projected map (see the "Model validation" section). Then, we simulated LULC maps for 2016, 2022, 2028, and 2034, starting from the observed 2010 LULC map. Finally, we performed the validation and analysis results by applying the Intensity Analysis.

### Model validation—intensity analysis

In this research, we used the Intensity Analysis technique to quantitatively examine the simulation performance of the FLUS model in the interval, category, and transition levels. Aldwaik & Pontius^28^ thoroughly detailed the procedure and equations of Intensity Analysis. At the interval level, the total size of the change and the annual rate of change is calculated for each time interval (i.e., for the pair of maps defining each time interval). This level of analysis allows us to identify intervals with slow and fast rates of annual change. At the category level, the size of gross gains and losses and the intensity of gross gains and losses are calculated for each land class. This level of analysis identifies dormant and active land types in a specific time interval. Finally, the size and intensity of land-type transitions are calculated at the transition level. For each land type with gains or losses, this level of analysis identifies other land types that are notable targets of transition and notably avoided in transitions.

We first validated the results from the ANN model. Then we validated the model by comparing the 2004–2010 reference LULC change with the projected 2004–2010 projected map. Finally, we compared the 1998–2010 observed LULC change with the projected 2016–2034 LULC change in terms of the interval, category, and transition intensities.

### ANN validation

We used the Area Under the Curve (AUC) of the Total Operating Characteristic (TOC) index to quantify the ANN model performance. The TOC calculates how the ranks of an index variable (probability-of-occurrence maps) classify between the presence and absence in a binary reference variable (observed values of LULC)^46^. The TOC shows misses, hits, false alarms, and correct rejections. As in the Receiving operating curve (ROC), the AUC of the TOC offers a metric to summarise the performance. The AUC is used as an indicator for overall prediction accuracy. Generally, AUC values go from 0 to 1, where values between 0.60 and 0.70 mean poor accuracy level, 0.70–0.80 indicates fair, 0.80–0.90 signifies good, and 0.90–1 means excellent^47^.

### Land-cover projection validation

Since the neighborhood effect parameter in the Cellular Automata (CA) model is essential during model calibration, we applied a Sensitivity Analysis (SA) process by varying the neighborhood with 3 × 3, 5 × 5, and 7 × 7 sizes. We calculated the Figure of Merit (FoM) to assess the SA. FoM is a rate where the simulated and reference change intersection is represented in the numerator, while the union of simulated and reference change is at the denominator^27^. Then, we followed the Intensity Analysis framework proposed by Aldwaik & Pontius^28^. We evaluated the performance of the modeled LULC map by comparing the reference change versus the predicted change in the study area. A simulation of LULC changes from 2004 to 2010 was run to test the model, and we contrast the result with the observed change from the 2004 to 2010 LULC maps. Lastly, we compared the 1998–2010 reference LULC^28^ change with the simulated 2016–2034 land-use change regarding its interval, category, and transition level.

### Software tools

We downloaded the FLUS model from https://www.geosimulation.cn/FLUS.html. Then, we carried out the analysis using various software packages. First, we calculated the cross-tabulation matrices using TerrSet^48^. Second, we use the statistical software RStudio 2022.07.0^51^ to obtain the AUC for the TOC curve using the packages "raster ^49^ " and "TOC" and the "lulcc” package to compute the FoM^52^. Third, we applied Intensity Analysis employing the free software from http://www.clarku.edu/~rpontius/ and the package “intensity analysis^50^″ for RStudio 2022.07.0^51^. Finally, we made the maps with ArcGIS Pro 2.6^37^.

## Results

### Land use and land cover (LULC) change

**Figure 4:**
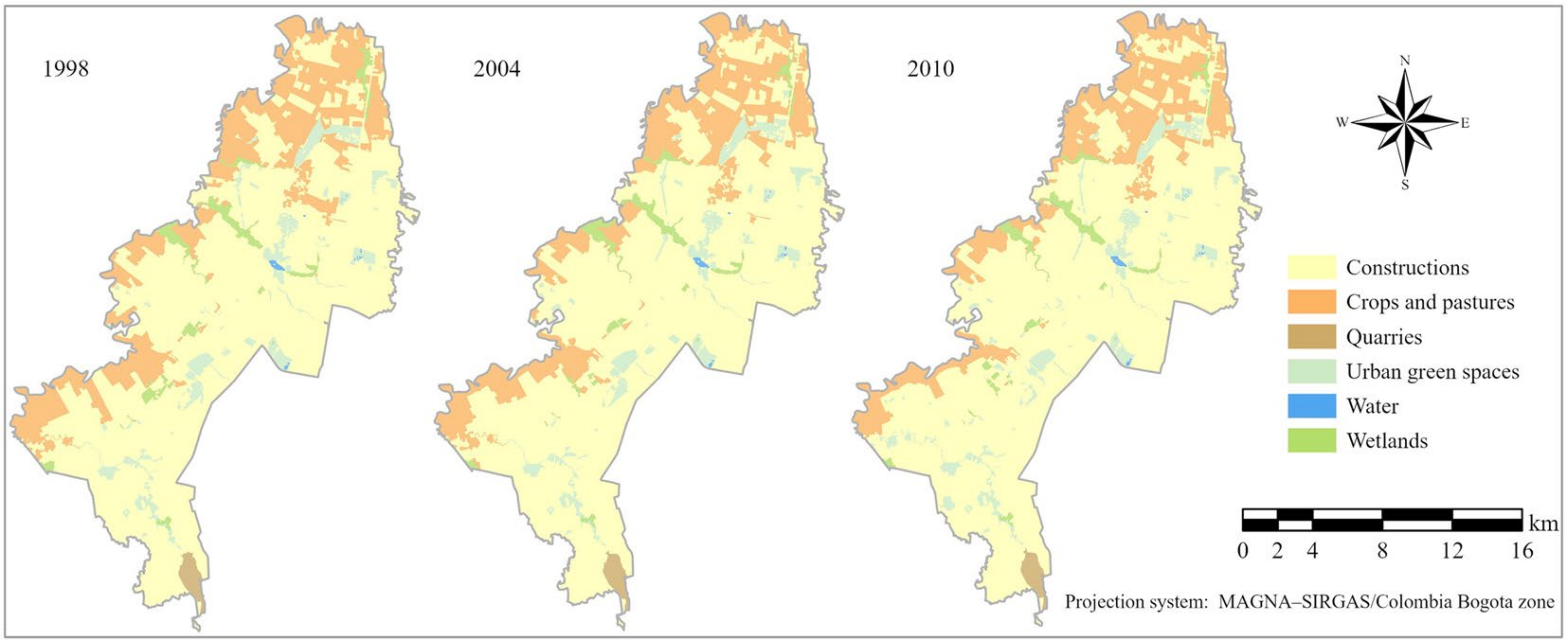
Maps of six land categories in 1998, 2004, and 2010.

Past LULC was calculated in three maps that included six classes, as follows: constructions, crops and pastures, quarries, urban green spaces, water, and wetlands (Fig. 4). Table 2 shows the results from comparing the three maps. Among the six land classes, the classes of urban green spaces, constructions, and water presented an increase in area. In contrast, the classes of crops and pastures, wetlands, and quarries decreased, with the crops and pastures showing the most significant decrease by 1211 ha less.

**Table 2:** Area (ha) per land class and relative area increment or decrement in 2010 compared with 1998.

	1998	2004	2010	Increment/decrement (%)
Constructions	21 849.4	22 544.7	22,995.3	5.2
Crops and pastures	6 709.9	5 996.9	5499.3	− 18
Quarries	254.1	243.9	244.3	− 3.9
Urban green spaces	1 592.1	1 659.5	1758.7	10.5
Water	136.8	136.8	137.9	0.8
Wetlands	810.2	770.7	717.0	− 11.5

Wetlands represented 2.58% of the total study area in 1998, 2.46% in 2004, and 2.29% in 2010, decreasing in 12 years by 0.3% of the study area. So then, the wetlands reduction from 1998 to 2004 represented a 4.87% loss in wetlands class area and 1.51% in 2010. These results show the continuous decrease of the wetland cover over the years. On the contrary, the area covered by constructions, representing the largest class in the study area, increased by 3.65% from 1998 to 2010, going from 69.7% in 1998 to 72% in 2004 and 73.3% in 2010.

### Validation results

#### ANN performance

We calibrated the FLUS model using LULC maps and driving factors and employed the model to obtain the projected maps for 2016, 2022, 2028, and 2034. Applying the ANN module, we generated the land transition probability map for each land type (see Supplementary Fig. S2). Those maps illustrate the chance of each cell transitioning to each of this study's six land types.

**Figure 5:**
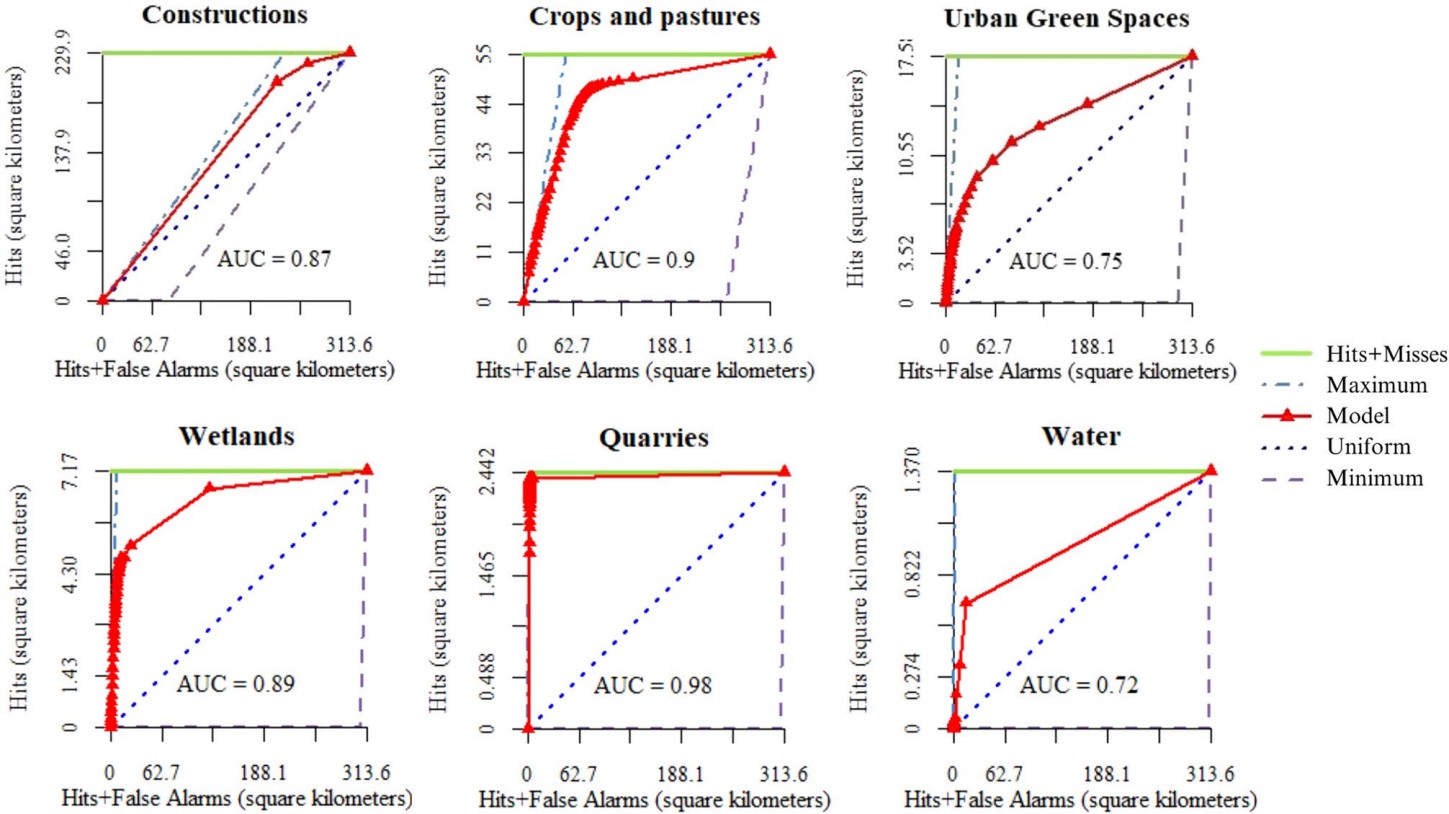
TOC curves and AUC values to validate the ANN.

Figure 5 shows the TOC curves of the six land types. The AUC values of each land class were computed according to the TOC curves. Validation of the ANN model showed the AUC to be above 0.7 in all land classes, with values ranging from 0.72 to 0.98 and a mean value of 0.85.

### LULC projection validation

At the interval level, results show that land change is quite fast for the first reference interval, 1998–2004, while land change is relatively slow for the second reference interval, 2004–2010. Table 3 shows LULC transitions in percentages during calibration and validation time intervals. During the 1998–2004 reference period, the overall reference change was 1617 ha. The overall reference change during 2004–2010 was 1480 ha, while during the 2004–2010 simulation, the overall change was 716 ha.

**Table 3:** LULC transition, each row is the start time and each column the end time. For each transition, the top number gives a 1998–2004 reference, the middle number gives a 2004–2010 reference, and the bottom number gives 2004–2010 simulation.

Category	Constructions	Crops and pastures	Quarries	Urban green spaces	Water	Wetlands	Total (%)	Loss (%)
Constructions	68.51	0.80	0	0.25	0	0.09	69.66	1.15
	70.66	0.78	0	0.36	0	0.08	71.87	1.22
	71.78	0.04	0	0.09	0	0	71.91	0.13
Crops and pastures	2.82	18.18	0	0.18	0	0.27	21.44	3.26
	2.08	16.54	0	0.40	0	0.15	19.17	2.63
	1.88	17.14	0	0.11	0	0	19.13	1.99
Quarries	0.03	0	0.78	0	0	0	0.81	0.03
	0	0	0.78	0	0	0	0.78	0
	0.02	0	0.76	0	0	0	0.78	0.02
Urban green spaces	0.21	0	0	4.85	0	0	5.08	0.23
	0.44	0	0	4.83	0	0.01	5.29	0.46
	0.02	0	0	5.27	0	0	5.29	0.02
Water	0	0	0	0	0.43	0	0.43	0
	0	0	0	0	0.43	0	0.43	0
	0	0	0	0	0.43	0	0.43	0
Wetlands	0.30	0.17	0	0.01	0	2.09	2.58	0.49
	0.13	0.26	0	0.02	0	2.04	2.46	0.41
	0.09	0.04	0	0	0.00	2.33	2.46	0.13
Total (%)	71.87	19.17	0.78	5.29	0.43	2.46	100	5.16
	73.31	17.58	0.78	5.61	0.44	2.29	100	4.72
	73.78	17.22	0.76	5.47	0.43	2.33	100	2.28
Gain (%)	3.37	0.99	0.00	0.44	0	0.36	*5.16*	
	2.65	1.04	0	0.77	0	0.24	*4.72*	
	2.01	0.08	0	0.20	0	0	*2.28*	
Net change (%)	2.22	2.27	0.03	0.21	0	0.13		
	1.44	1.59	0	0.32	0	0.17		
	1.87	1.91	0.02	0.18	0	0.13		

*The underlined numbers represent the percentage of total LULC gained during the studied time intervals.

We applied the SA to see how the size of the neighborhood influences the results. First, the simulation map showed that the change was allocated around the edges of zones of certain land types, as seen in Supplementary Fig. S3. In contrast, the change in the reference maps is rarely allocated on those edges. Next, we calculated the FoM to evaluate SA results. FoM values were 7.08% for 3 × 3 neighborhood size, 7.14% for 5 × 5, and 7.09% for 7 × 7. A wide variety of metrics were implemented to evaluate the SA of the model, and we found that the accuracy of the outputs model improves with a 3 × 3 neighborhood size and 5 m spatial resolution. For a detailed explanation of SA results, see Cuellar & Perez^51^.

In Supplementary Figure S4, we presented the results from the Intensity Analysis at the category level for each time interval. The dashed line in each graph illustrates the uniform intensity of annual change. The analysis showed a deceleration of the overall change from the first-time interval (1998–2004) to the second-time interval (2004–2010), with the simulation deceleration being more intense than the reference downtrend. Supplementary Figures S4a and S4c show that the 2004–2010 simulation loss intensities are less than the 1998–2004 reference loss intensities. Additionally, Supplementary Figures S4a and S4b depict that the reference patterns are not stationary from the calibration interval (1998–2004) to the validation interval (2004–2010). Notably, the reference wetland category gains more during the calibration interval than during the validation interval.

Consequently, this category’s intensity in the simulation does not match the reference from 2004–2010. Comparing Supplementary Figure S4b and S4c reveals more information about model performance in the validation time interval (2004–2010). While Reference change shows gains and losses in categories, in the simulation, categories either gain or lose. In other words, in the simulation, the relative difference between the gain and loss of each category is significant, except for the water category, which shows very little change. It is also noteworthy that in reference data of calibration and validation time intervals, the most extensive loss is in wetlands, and the second largest is in crops and pastures. In contrast, the most significant loss in simulation is in crops and pastures, and the second largest is in wetlands.

Supplementary Figure S5 presents the transition-level results for wetlands, crops and pastures, urban green spaces, and constructions. Comparing the calibration and validation time intervals shows how the model almost extrapolated change intensities from the 1998–2004 reference calibration interval to the 2004–2010 simulation validation interval. The gain of wetlands targeted crops and pastures loss during all the intervals. However, unlike reference change in the validation interval, it did not target urban green space loss in the simulation. The construction gain targeted crops and pastures in all intervals and targeted wetland loss during the calibration interval. In contrast, gains of urban green spaces, crops, and pastures from all other categories are stationary across the three intervals.

### LULC change intensity analysis

#### Interval level change

The annual change intensity for all land classes is 0.16% between 1998 and 2034; this decreases from the historical (1998–2010) to the future (2010–2034), 0.63% and 0.25%, respectively. However, the rate of change in the study area was not uniform. For example, the annual change intensity in 1998–2010 was *fast*, whereas the projected intensity in 2010–2034 was *slow*.

#### Category level change

**Figure 6:**
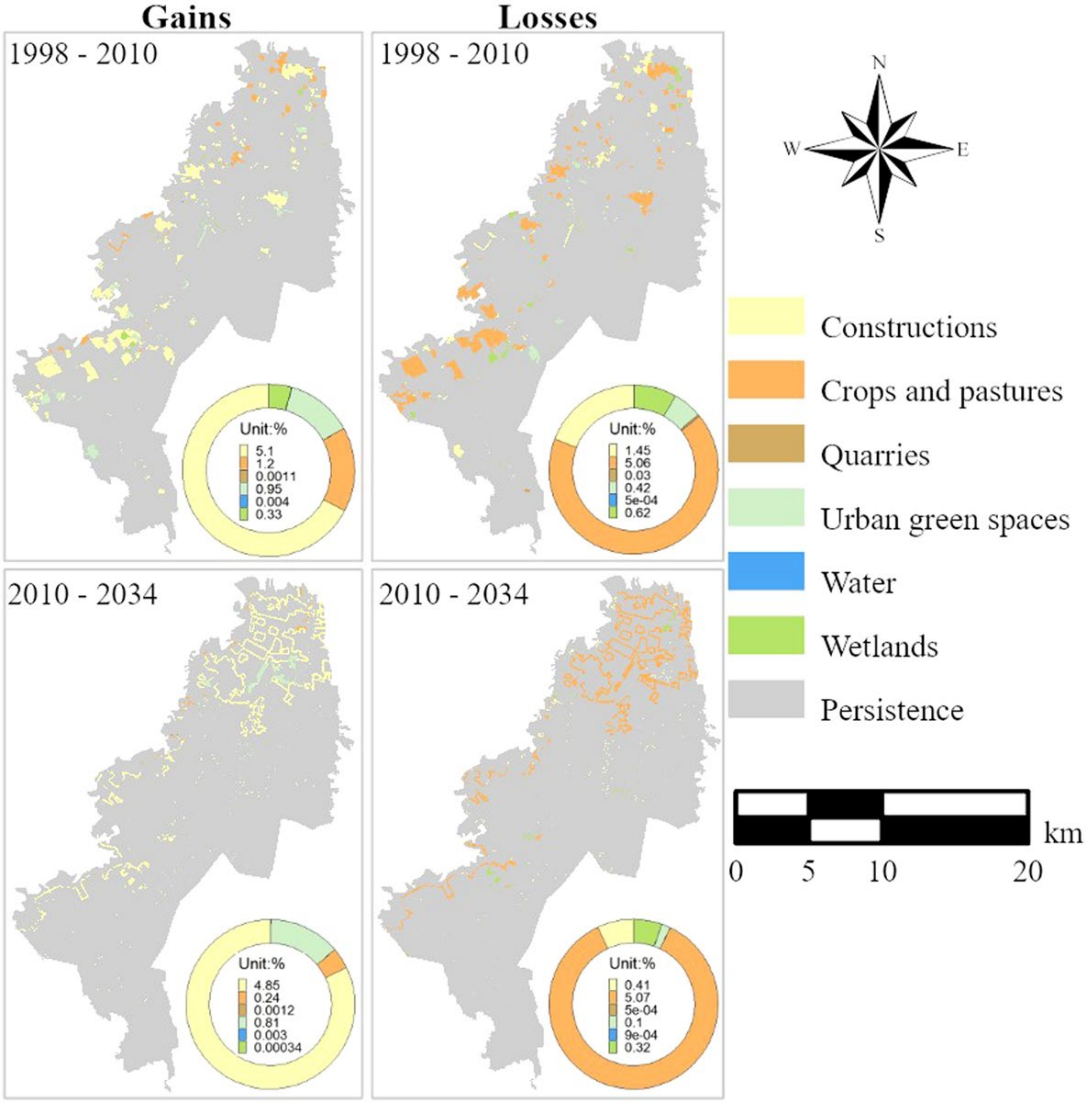
Gains and losses of each land class in 1998–2010 and 2010–2034.

The wetlands, constructions, crops and pastures, and urban green spaces categories had more significant changes than other land categories (Fig. 6). In 1998–2010, gains in construction land class were principally in the west of the study area, while losses were mainly in the north. In contrast, the gains and losses in 2010–2034 are distributed across the study region. Historical (1998–2010) and projected (2010–2034) gains in crops and pastures were mainly in the north, though the projected gains are much less than the historical gains. In comparison, losses in this category were distributed in the west from north to south from 1998–2010. The 2010–2034 losses are concentrated in the north, with some other losses near the western border of the study area. Finally, the 1998–2010 gains for wetlands are settled mainly in the northwest area, while losses are in the center and south. This category's projected gains are negligible, and projected losses are distributed in the center and north of the study area.

**Figure 7:**
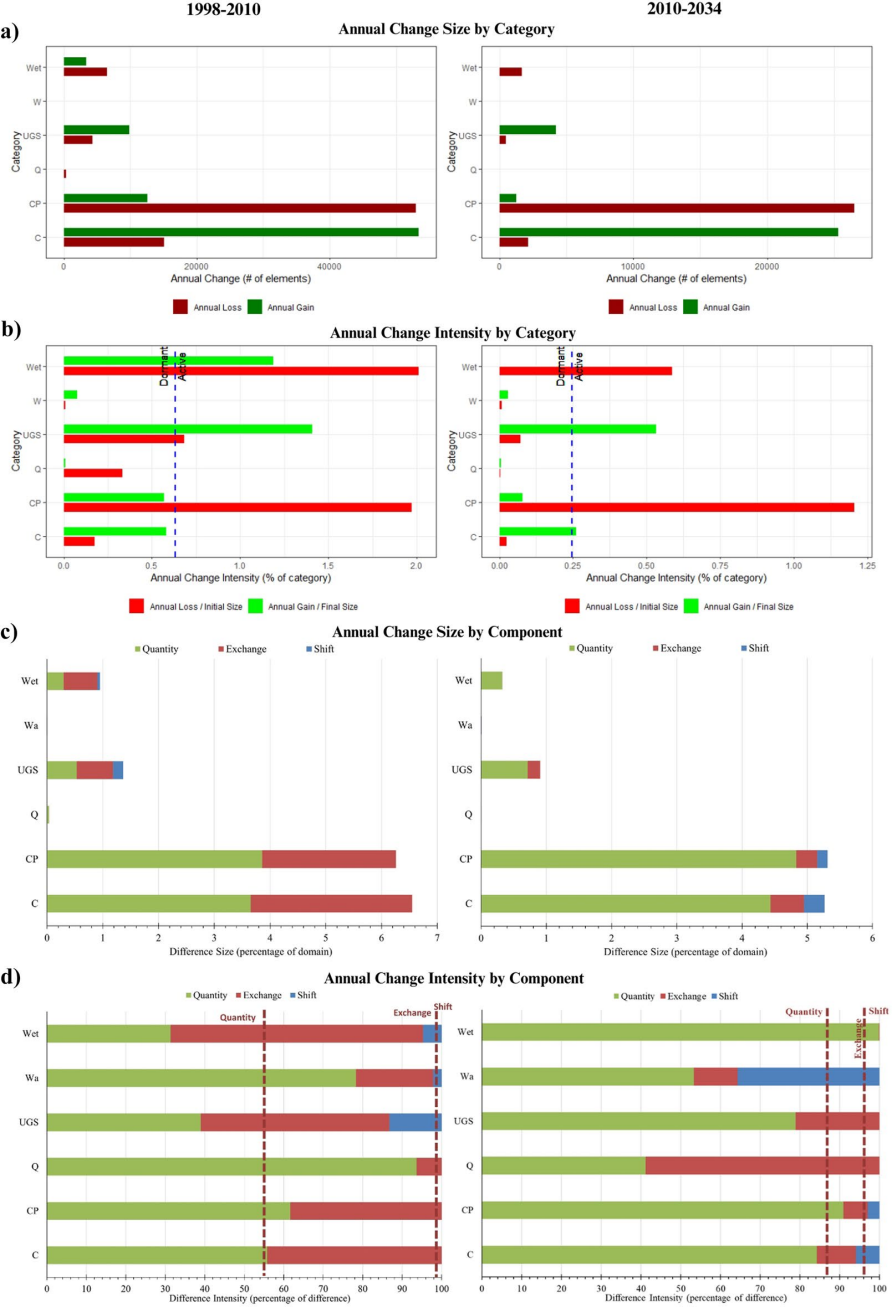
Category Level Intensity Analysis. Rows (**a**) Land change size gains and losses, (**b**) land change intensity, (**c**, **d**) change size and intensity by components. Wetlands (Wet), urban green spaces (UGS), crops and pastures (CP), quarries (Q), water (Wa), and constructions (C).

In 1998–2010, the constructions, crops, and pastures experienced the most significant coverage change compared to the other land categories. The same behavior was projected in 2010–2034 (Fig. 7). Furthermore, in Fig. 7a, constructions exhibited a noticeable gain during both periods. In contrast, losses were primarily noticeable in crops and pastures. Although the gain and loss of wetlands are small in both intervals, their annual loss intensity is much higher than that of constructions and higher than crops and pastures in 1998–2010. In the projected period (2010–2034), wetlands loss intensity is much higher than construction gains and loss intensities (Fig. 7b). For crops and pastures, its annual gain intensity was *dormant* over both time ranges (Fig. 7b), but its loss intensity was higher than that of the wetlands in 2010–2034. The urban green spaces category was the most *active* during both intervals regarding annual gain intensity. Wetlands' gain intensity was *active* during 1998–2010 and negligible in 2010–2034, but its loss intensity was *active* in both periods.

**Figure 8:**
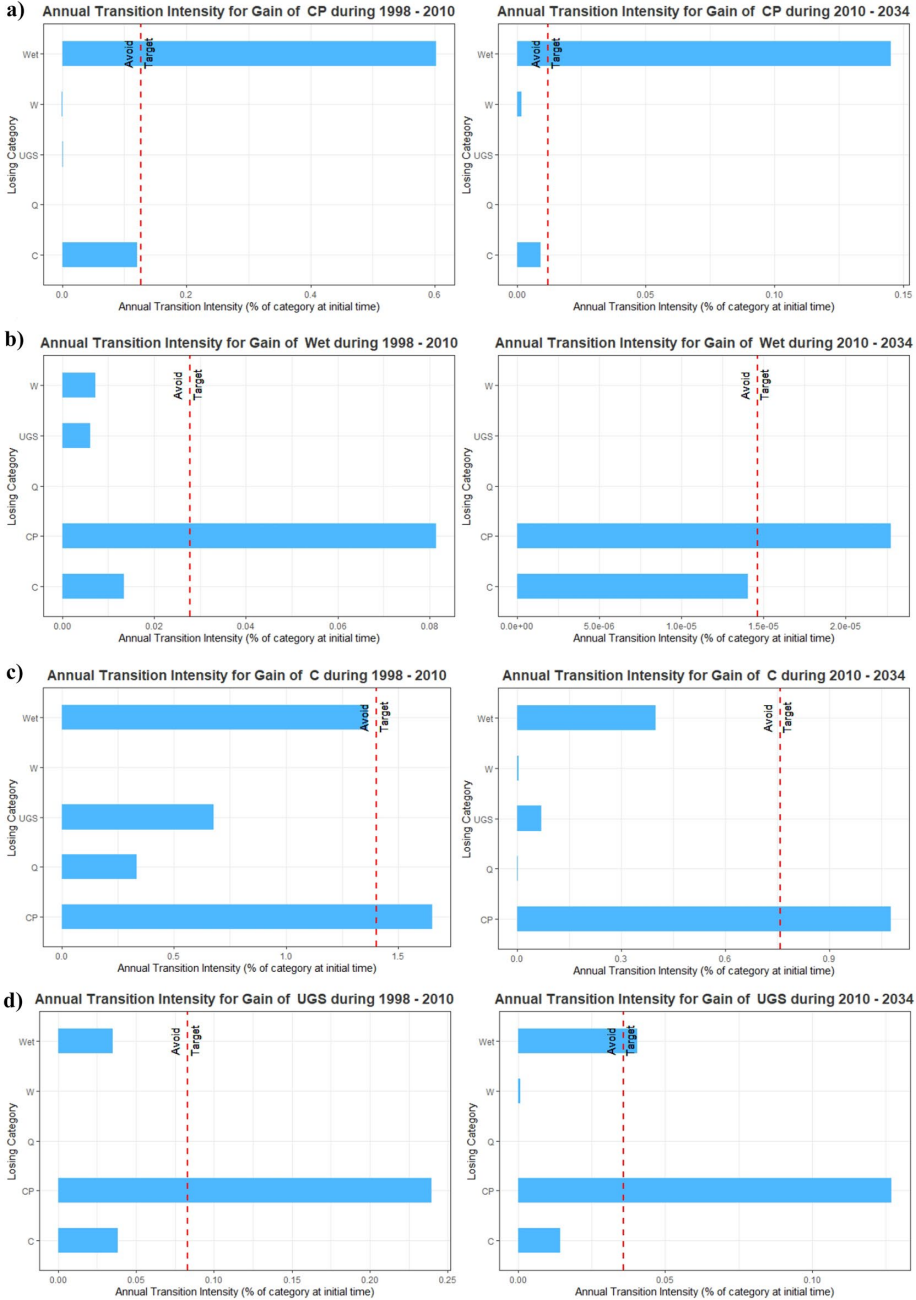
Transition intensity from losing (**a**) crops and pastures, (**b**) wetlands, (**c**) constructions, (**d**) urban green spaces, and land classes during 1998–2010 and 2010–2034. Wetlands (Wet), urban green spaces (UGS), crops and pastures (CP), quarries (Q), water (Wa), and constructions (C).

Figure 7c shows crops and pastures, and constructions had the most considerable differences in both time intervals. Their *quantity* component is larger than the other two intensity components. Both are more intensive than *quantity* in 1998–2010, but crops and pastures are more intense than *quantity* in 2010–2034. Urban green spaces and wetlands do not have a minor *quantity* component, but they were the only ones with a *quantity* component less intensive than the *quantity overall* in 1998–2010*.* In contrast, in the projected period, the *quantity* component is minor for wetlands in size but more intensive for wetlands than for the other categories. Urban green spaces and wetlands have the most *significant exchange* component*,* and both are more intensive than *exchange overall in 1998–2010.* In 1998–2010, the only categories that had shift were wetlands, urban green spaces, and water, and all are more intensive than *shift overall.* Whereas in 2010–2034, constructions, crops and pastures, and water are the only categories that have a *shift,* just constructions and water are more intensive than *shift overall.* The overall quantity line in Fig. 8d indicates that *quantity* is 55% of the difference in all six categories. *Exchange* accounts for 43% of the difference overall, and *shift* accounted for 1% in 1998–2010. In contrast, in the projected interval, those intensity components are 87% in *quantity*, 9% in e*xchange*, and 4% in the s*hift.*

### Transition level change

Figure 8 shows the results of the transition-level Intensity Analysis for the most significant gains: wetlands, crops and pastures, constructions, and urban green spaces. Although during the time intervals of 1998 to 2010 and 2010–2034, the transition intensities from crops and pastures to constructions and wetlands are more substantial than that from the remaining categories, from those results, it can be observed that the gain of constructions and wetlands comes more from the loss of crops and pastures. The transition intensities from wetlands, crops, and pastures to urban green spaces differ over the two periods. In 1998–2010, the gain of urban green spaces *avoid*ed the loss of wetlands but *targeted* the loss of crops and pastures, while it *targeted* losses in both categories during 2010–2034. The gain of crops and pastures *targets* the loss of wetlands in both time intervals and *avoids* the loss of constructions.

Additionally, the gain of crops and pastures, constructions, and wetlands is stationary through time to how those categories *avoid* or *target* the non-crops and pastures, non-constructions, and non-wetlands categories. However, the gain of urban green spaces is not stationary over time. For example, the gain of urban green spaces *targeted* only crops and pastures during 1998–2010, but during the projected period, it also *targets* wetlands areas.

Similarly, the annual transition intensity indicated the strength of the transitions and revealed that constructions targeted crops and pastures during both periods. As a result, vast gains in constructions from crops and pastures during the study spans were not initially caused by this large area but rather by the construction gains causing this category to lose area intensively. The second category, which gained annually in size, was crops and pastures; its gains came from wetlands. In addition, the annual transition intensity indicated the strength of the transitions and revealed that crops and pastures targeted wetlands in both time intervals. Consequently, a considerable gain in crops and pastures from wetlands was not caused by its area at the initial time but rather by the gains in crops and pastures causing this category to lose area intensively. Finally, wetland gains were observed from crops and pastures, as well as urban green spaces. It hinted at the power of the transitions and revealed that these categories of lands targeted crops and pastures in both periods too.

### Future simulated LULC change in quantity

**Figure 9:**
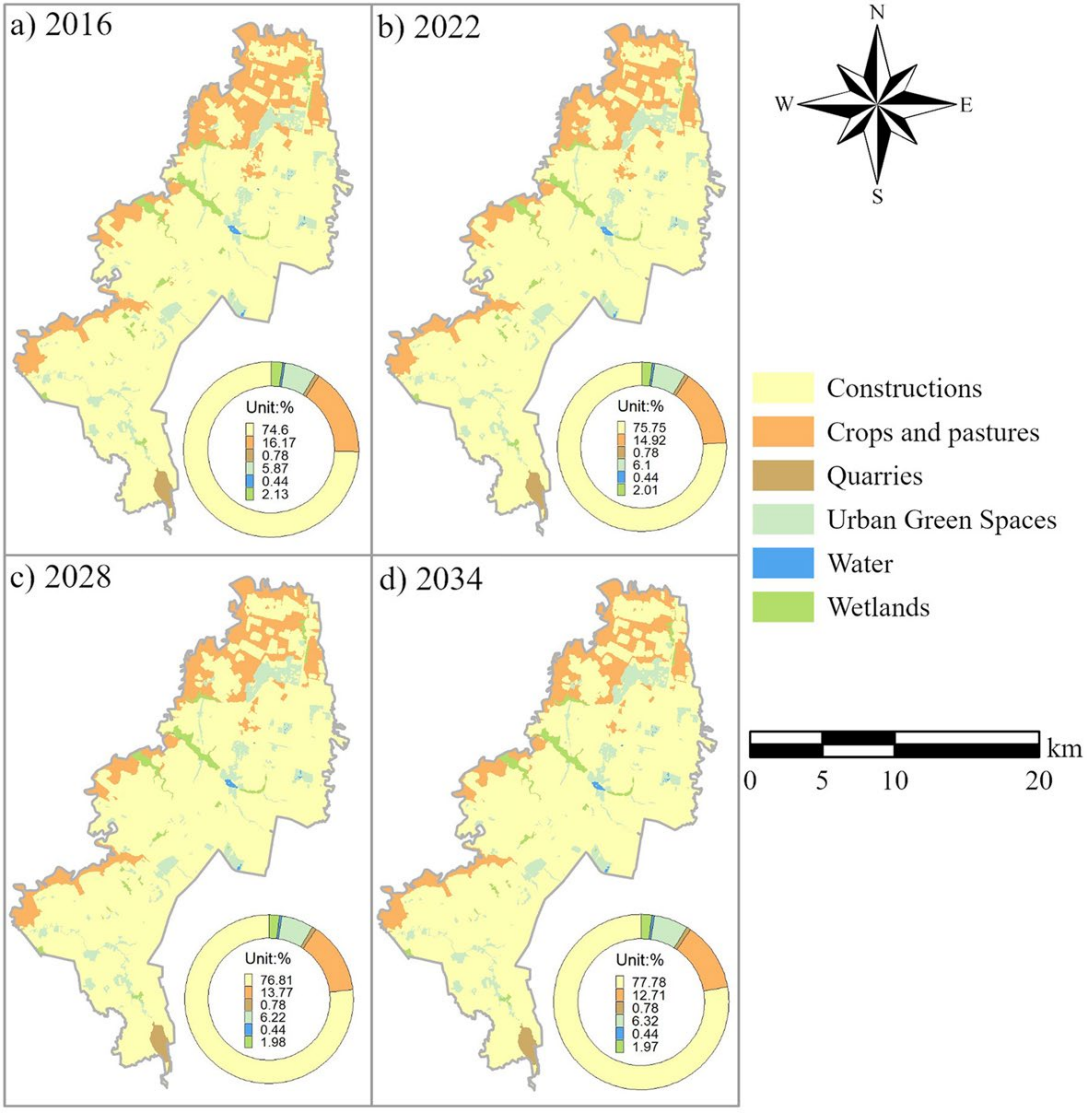
Percentage (concerning the total area) of projected LULC in (**a**) 2016, (**b**) 2022, (**c**) 2028, and (**d**) 2034.

Figure 9 shows the projected LULC maps. The cultivated land was mainly in the north and west of the study area bordering the Bogota River. Its coverage declined from 21.4% in 1998 to 12.71% in 2034. The wetlands land was principally found in areas connected with watercourses, declining from 2.6% in 1998 to 1.97% in 2034. The urban green spaces were located throughout the study area, and their coverage increased from 5% in 1998 to 6.3% in 2034. The constructions represented a vast extent in the study area, and their coverage grew from 70% in 1998 to 78% in 2034.

Constructions, crops and pastures, and urban green spaces categories of land are the three LULCs that dominate the study area and comprise about 96% of the total (Table 4). During 1998–2010, there were net changes of 3.86% for the crops and pastures, for the constructions and green spaces 4.18%, for water and wetlands 0.30%, while for water and quarries, it was near-zero. Land change assessed at the general landscape level showed that construction was the leading land-gaining category after urban green spaces. Crops and pastures cover was the main losing category. The gains in construction and urban green spaces were 1600 ha and 297 ha, respectively. Constructions lost approximately 454 ha, but crops and pastures lost the most with 1587 ha, nearly the same that the constructions category gained. Wetlands gained 102 ha and lost approximately 195 ha, with a net change of almost 93 ha. Likewise, the simulation results showed a similar pattern for all the land covers throughout the study area. The output map for the year 2034 shows a decrease of almost 0.33% in wetlands coverage concerning the extension in 1998, which translates into 101 ha.

**Table 4:** LULC transitions in 1998–2034 based on comparing the reference LULC in 1998 and the projected LULC in 2034. Each row is the start time, and each column is the end time. For each transition, the top number gives the 1998–2010 reference, and the bottom provides the 2010–2034 simulation.

Category	Constructions	Crops and pastures	Quarries	Urban Green Spaces	Water	Wetlands	Total (%)	Loss (%)
Constructions	68.21	1.01	0	0.32	0	0.11	69.66	1.45
	72.93	0.16	0	0.25	0	0	73.35	0.41
Crops and pastures	4.23	16.38	0	0.62	0	0.21	21.44	5.06
	4.53	12.47	0	0.53	0	0	17.54	5.07
Quarries	0.03	0	0.78	0	0	0	0.81	0.03
	0	0	0.78	0	0	0	0.78	0
Urban Green Spaces	0.41	0	0	4.66	0	0	5.08	0.42
	0.10	0	0	5.51	0	0	5.61	0.10
Water	0	0	0	0	0.43	0	0.43	0
	0	0	0	0	0.44	0	0.44	0
Wetlands	0.42	0.19	0	0.01	0	1.96	2.58	0.62
	0.22	0.08	0	0.02	0	1.97	2.29	0.32
Total (%)	73.31	17.58	0.78	5.61	0.44	2.29	100	7.58
	77.78	12.71	0.78	6.32	0.44	1.97	100	5.90
Gain (%)	5.10	1.20	0	0.95	0	0.33	*7.58*	
	4.85	0.24	0	0.81	0	0.0003	*5.90*	
Net change (%)	3.65	3.86	0.03	0.53	0	0.30		
	4.44	4.83	0	0.71	0	0.32		

*The underlined numbers represent the percentage of total LULC gained during the reference and simulated time intervals.

Meanwhile, constructions and green spaces cover continue their pattern with a net gain of 5.15%, that is 1614 ha. On the other hand, the construction class continues to record the highest losses with 1589 ha less. In other words, based on the reference data, by 2034, the total area of constructions and urban green spaces will be equivalent to 26,358 ha, for wetlands almost 616 ha, and 3982 ha for crops and pastures.

### Future wetland changes in Bogota

**Figure 10:**
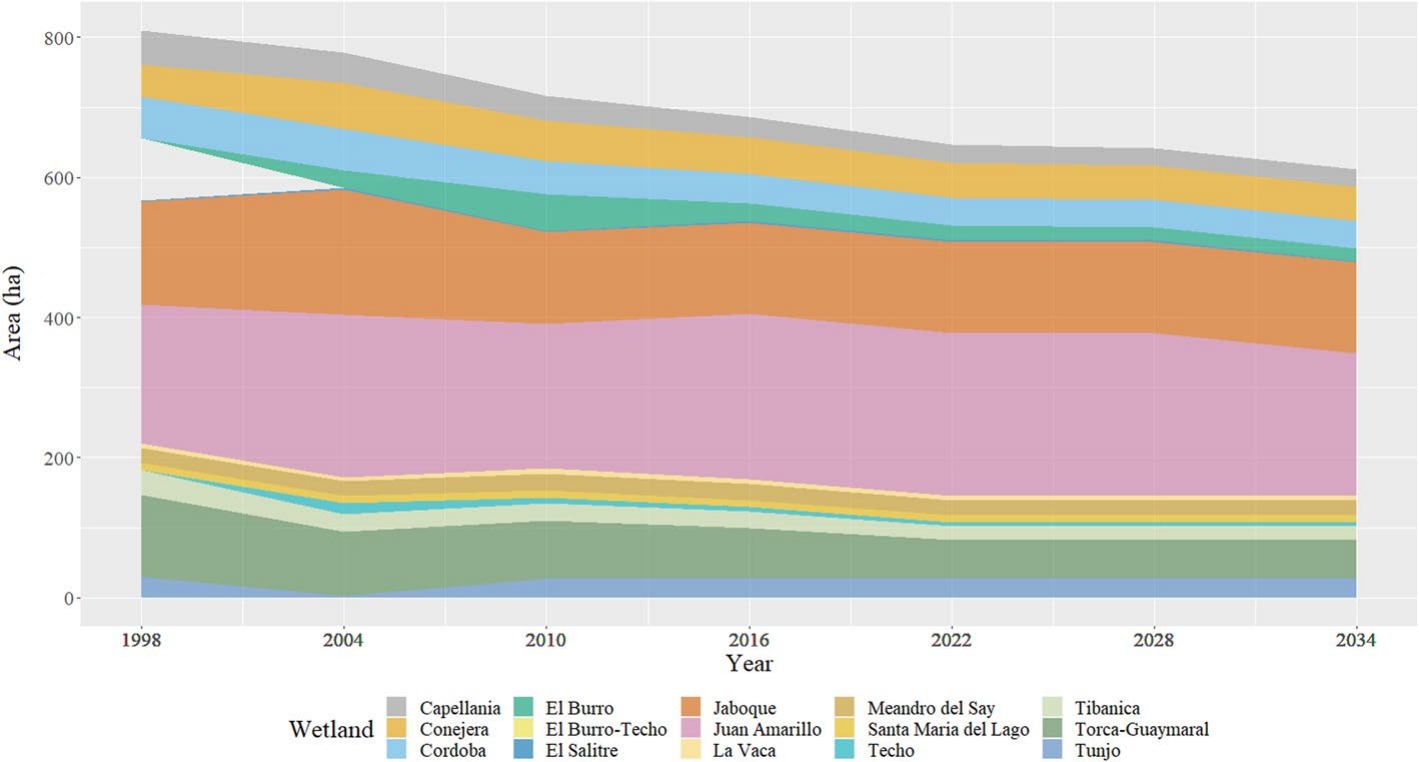
Wetlands areas from 1998 to 2034.

In this research, we utilized the FLUS model to obtain the projection of the spatial distribution of Bogota’s wetlands. To explore their spatiotemporal changes, we calculated their areas from 1998 to 2034 (Fig. 10). The reference pattern indicated an increase in the northeastern wetlands, except for Torca-Guaymaral, Jaboque, and Cordoba, which had a decreasing pattern. In addition, the wetlands located in the southwest indicated a decreasing reference pattern, except for La Vaca, which had an increasing pattern. Finally, El Salitre and Santa Maria del Lago wetlands maintained a similar pattern during the reference period. Therefore, the simulation maps founded on the spatial dynamics during the historical time 1998–2010 displayed a similar pattern in the study area. The simulated map for the year 2034 shows an increase in 3% of the wetlands located in the northwest, except for Torca-Guaymaral, Jaboque, and Cordoba, with a 52%, 11%, and 34% decrease, respectively. Also, it shows a reduction in 54% of the southwestern wetlands, except for La Vaca, with a 6% decrease. To sum up, by 2034, the total wetlands area is projected to be 611 ha, losing almost 25% of its surface compared to 1998.

## Discussion

Urban wetlands provide numerous ecosystem services, such as reducing urban heat island effects, providing bird shelter and passive recreation areas, and enhancing water quality^2^. However, urban wetlands remain highly vulnerable to human intrusion. The main drivers of the degradation of urban wetlands are population increase, uncontrolled urban development, water pollution, land use change, drained water, water regime change, and loss of biodiversity^4^. Worldwide, and especially in the twenty-first century, the natural habitat and landscape layout have been drastically modified. Changes in LULC have been the subject of multiple investigations over the past two decades^45,52–54^. Using spatiotemporal data, we analyzed LULC changes in the area of influence of the Bogota wetlands from 1998 to 2010. From the LULC maps and the physical and socioeconomic determinants, we obtained the probabilities of occurrence of each LULC using the ANN of the FLUS model. In addition, we obtained LULC projections for 2016, 2022, 2028, and 2034 using the hybrid ANN-Markov-CA FLUS model.

We computed the AUC values of each land class according to the TOC curves to evaluate the ANN. Then, the TOC compared the simulated map with the reference map in 2010. For all LULC types, the AUC was above 0.7, indicating a high degree of agreement between the maps. Figure 5 shows that the spatial uniformity between the reference and simulated map for wetlands is significantly increased by 89%. Then, the AUC values indicated that the adjustment of the probability of occurrence of the individual LULC, obtained from the training of the ANN, can be well explained by the selected driving factors.

During the validation, sensitivity analysis showed that all neighborhood configurations caused the simulated changes to occur near the sides of the patches (see Supplementary Fig. S3). SA results show that different neighborhood sizes cause the gain of a ground cover to be around patches of existing ground cover, as it turned out to be in previous research such as that of Varga et al.^27^. The FoM components indicated that the simulation change only corresponds partially to the reference change in any neighborhood size. Misses were greater than false alarms, which means that reference change from 2004–2010 was more significant than that simulated by the model. Additionally, the Intensity Analysis's validation at the interval level displayed that the FLUS model adequately simulated the decline of global change from the calibration period (1998–2004) to the validation period (2004–2010). At the category level (see Supplementary Fig. S4), it showed that the model simulated the active or dormant form of loss or gain of each LULC with almost the same state of the LULCs during the calibration interval, except for the constructions that during the calibration period was dormant and became active in the validation period. At the transition level, the results showed that the simulated gain of crops and pastures targeted wetlands, a stationary pattern during time according to the reference data. Also, the transition level showed that the simulated increase of constructions targeted wetlands decreased during the calibration interval; however, during the validation interval, the expansion in constructions just targeted crops and pastures (see Supplementary Fig. S5). So, the pattern for construction was not stationary.

Validation analysis showed that the study area has a vast dormant category phenomenon. Constructions land class accounts for most of the extent domain in the historical period (Table 3); it is dormant regarding gains and losses (Supplementary Fig.S4). Also, results showed that losses in each LULC are mainly caused by the gain in constructions (Supplementary Fig. S5). Nevertheless, gains in crops and pastures land class were the leading cause of wetlands land class loss (see Table 3 and Supplementary Fig. S5). Then, the large extent of construction land use performs an essential role in the total change because the reference change is 5.16% of the entire study area, and nearly 4.5% involves transitions with construction (see Table 3). A similar phenomenon was reported in the Indonesian forest, where the large forest size played an essential role in the gains and losses behavior of the bare and grass categories^55^.

We employed the Intensity Analysis approach to analyze the LULC change in 2010–2034 in terms of its interval, category, and transition level, focusing on the wetlands' category. Mainly construction class increased primarily in the historic interval and likely will continuously increase by 2034. The pattern of urban expansion is evident towards the western part of the city, areas occupied mainly by agricultural activities but which, with the establishment of new (primarily illegal) neighborhoods further south in Bogota, progressively converted its LULC from crops and pastures to constructions. Similar patterns were found by Czerny & Cezerny^56^. This phenomenon was portrayed in the intensity analysis at the transition to the construction level (see Fig. 8). The intensity analysis showed that crops and pastures category coverage has been and will be the most affected globally by other coverages and constructions that go hand in hand with the creation of new green areas and wetland coverage. Rashid and Aneaus^57^ found that agricultural areas decline mostly by built-up areas in the urban wetland in Kashmir Himalaya, India. Although the dynamics of LULC change from crops to constructions/green areas are mainly due to recent human settlements, the role of wetlands needs to be analyzed in more detail.

Simulation maps based on the spatial dynamics during the historic time 1998–2010 displayed a similar pattern for wetlands cover. On the one hand, wetlands in the northwest had a reference pattern of expansion, except for Torca-Guaymaral, Jaboque, and Cordoba, which had a decreasing pattern. On the other hand, the wetlands located in the southwest indicated a decreasing reference pattern, except for La Vaca, which had an increasing pattern. In contrast, El Salitre and Santa Maria del Lago wetlands keep the same pattern during the reference period.

For La Conejera, we found that the increase in wetlands leads to a loss of green areas and crops, while it is a projected loss in construction. Likewise, more wetland area is projected to be lost due to crop expansion. These results are consistent with the last report of the District Secretary for Environment^58^, where there was evidence of an increase in crops by private farms located in the northern sector of the wetland. Juan Amarillo wetlands also had a pattern of expansion, which triggered a loss in crops and pastures land in the reference time but will target loss in construction. In addition, the presence of livestock in the wetland may be linked to its use as a livestock grazing area, a problem that has been the subject of attention in improving the ecosystem^59^. A similar behavior was evidenced in the Meandro del Say wetland, in which gains in wetland areas target the loss of crop areas, and gains in green areas target loss in the wetland surface. This behavior could be explained due to one of the stressors currently facing the wetland since large soccer fields in much of the wetland's interior belong to the El Say farm, where a soccer team trains^60^. Chang et al.^61^ found similar results in China under a scenario of protected farmland where the wetland area decreased rapidly compared with other land classes.

Regarding the wetlands that are projected to lose surface area, let us start with Torca-Guaymaral. A loss is projected due to the gain in crop cover. Although, its loss was also targeted by gains in construction in the historic period. Recent reports indicate agricultural activities in the wetland's ecological corridor^62^. On the part of the Jaboque wetland, in the past, its pattern was one of loss due to the gain of cultivated area. However, although the loss of its surface area is projected (Fig. 10), its intensity of loss is insignificant since the intensity analysis did not show an active loss of its cover due to the gain of another category. Despite the projections in this wetland, the model could not capture the spatiotemporal dynamics because the current stressors of the wetland do not match the results, given that the southern part of the wetland has been primarily affected by the creation of orchards^63^. In the Cordoba wetland, the observed dynamic was the loss of the wetland area due to the gain in construction land in both periods studied. Despite being one of the wetlands where citizen participation has been crucial to its recovery in the mid-2000s^64^, illegal occupation of the wetland space has caused impacts on the ecosystem because it has been used for improper activities that disrupt the habitat^65^. This pattern is projected to continue impacting its conservation.

Regarding the wetlands in the southern part of the study area, Capellania had the same loss pattern due to the gain in construction land class. Constructions cover has been and will continue to be an essential component in the fragmentation of the wetland since, for example, the Avenida la Esperanza passes through the wetland, dividing it into a northern and southern sector. In addition, the Avenida Longitudinal de Oriente is expected to pass through the wetland^66^. Towards the middle of the 1950s, the wetlands of the locality of Kennedy; La Vaca, El Burro, and Techo counted about 98 ha^67^. In the 1998 map, it is noted how these ecosystems began to be impacted by urbanization, transforming them into what is now known as the three ecosystems. Of this significant wetland, in 2010, it had lost at least 33% of its 1998 extension, and it is projected that its degradation will continue with a loss of up to 73% in 2034. In the southern zone of Bogota, the predominant characteristic of wetland conversion was due to the gain of building cover. These results are supported by research that shows that informal urbanization activity in sectors such as Kennedy gained strength in the locality to satisfy housing demands in the 1970s^67^. The problem of illegal settlements is not a matter of the past; the recent report of the District Secretary for the Environment reports new constructions in the non-legalized neighborhood of Lagos de Castilla^68^.

The information presented in this article focuses on the dynamics of change that have occurred in the urban wetlands of Bogota and puts into perspective how these patterns may continue to be replicated in the coming years. Adopt Intensity Analysis as a method of analyzing the results, which considers the intensity of each category's gross loss and gross gain concerning the temporal change overall; let us highlight exciting phenomena. First, it is evident that despite establishing a public policy on wetlands approximately twenty years ago, the results show that these ecosystems have continued to be affected by poor urban planning in the city. Sizo et al.^69^ obtained similar outcomes where the future conditions of urban wetlands in Saskatoon did not improve because of the lack of the historical trend of wetland loss and the conservation strategy. Contrary to popular belief, the results yielded interesting information regarding the stressors in each wetland studied. We found that these ecosystems have changed their nature to serve as shelter for the population and have been affected by using crops or pastures. At a global level, another remarkable aspect is the predominant loss of ​​crops and pastures historically located towards the area surrounding the Bogota River. We show that crops and pasture areas have been reduced due to the conversion to build sites jointly with green spaces.

Alongside the main findings of this study, it is important to mention its limitations. The first aspect to mention should be the level of error and/or uncertainty that can be introduced due to the nature of the input datasets and the manipulation they undergo; transformation, such as the vector to raster conversion and raster resampling, could influence simulation outcomes^70^. First, our land maps were converted from a vector to raster format, with a 5 m spatial resolution, given that in the sensitivity analysis, the results obtained at this resolution were more in agreement with the reference data. Second, simulating long-term land cover changes is complex due to the influence of diverse climatic, demographic, socioeconomic, and physical factors. In our study, we selected ten factors. However, we should have included future climate data, which, under the climate change perspective, will significantly benefit our understanding of the future dynamics of urban wetlands under climate change scenarios^71^. Third, data limitations made it impossible to conduct a study over a more extended period, and other recently declared wetland ecosystems, such as La Isla, Hyntiba-Escritorio, and Tingua Azul, were not included. We call on geographic information holders to facilitate open data with more unrestricted access. Fourth, the methodology adopted did not include vertical urban densification^72^. However, as many aspects of human welfare, for example, ecosystem services offered by these natural resources, are associated with housing density^73^, future research should include this variable to determine whether vertical densification changes impact wetland ecosystem services, especially its habitat quality.

## Conclusions

Wetlands' LULC reduction patterns from 1998 to 2010 will likely continue by 2034 despite being protected areas with restricted use. The greatest threat to wetlands was a human disturbance by construction activity or conversion to crop or livestock areas. It is assumed that it will continue to be so in the predicted time frame. However, the characteristics of wetland decrease in the reference and projected period differ. From 1998 to 2010, the northwestern urban wetlands decreased more due to crop conversion, while the wetlands in the south were more threatened by the expansion of building areas. Even though the Capital district's wetlands policy was established in 2005, the city's urban wetlands have continued to be subject to anthropogenic degradation. Then, the existing norms about their conservation and protection should be revised to ensure integrated management of that urban ecosystems.

It is time to become aware, to restore the value of these ancestral ecosystems, and to move from a preservationist vision to a conservationist one, where we protect, recover, and conserve the wetlands that provide so many benefits to the community and that will help us mitigate climate change.

## Supplementary Information

Supplementary Information.

## Data Availability

The datasets generated and/or analyzed during the current study are available in the open GitHub repository https://github.com/yacuellar94/Data-for-article-2-Yenny-Cuellar-Liliana-Perez.git.
