# Supplementary material for: Multitemporal modeling and simulation of the complex dynamics in urban wetlands: the case of Bogota, Colombia
Source: Sci Rep. 2023 Jun 9;13:9374. doi: 10.1038/s41598-023-36600-8 (PMC10256738; doi:10.1038/s41598-023-36600-8)
Supplement: Supplementary file 1 — Supplementary Information. [file 41598_2023_36600_MOESM1_ESM.pdf]

**Supplementary Material file**

**Multitemporal Modeling and Simulation of the Complex Dynamics in Urban Wetlands – The Case of Bogota, Colombia**

Yenny CUELLAR[a] and Liliana PEREZ[b]

Laboratoire de Géosimulation Environnementale (LEDGE), Département de Géographie, Université de Montréal, Montréal, QC H2V 0B3, Canada
Email addresses: a) yenny.cuellar@umontreal.ca, b) l.perez@umontreal.ca.

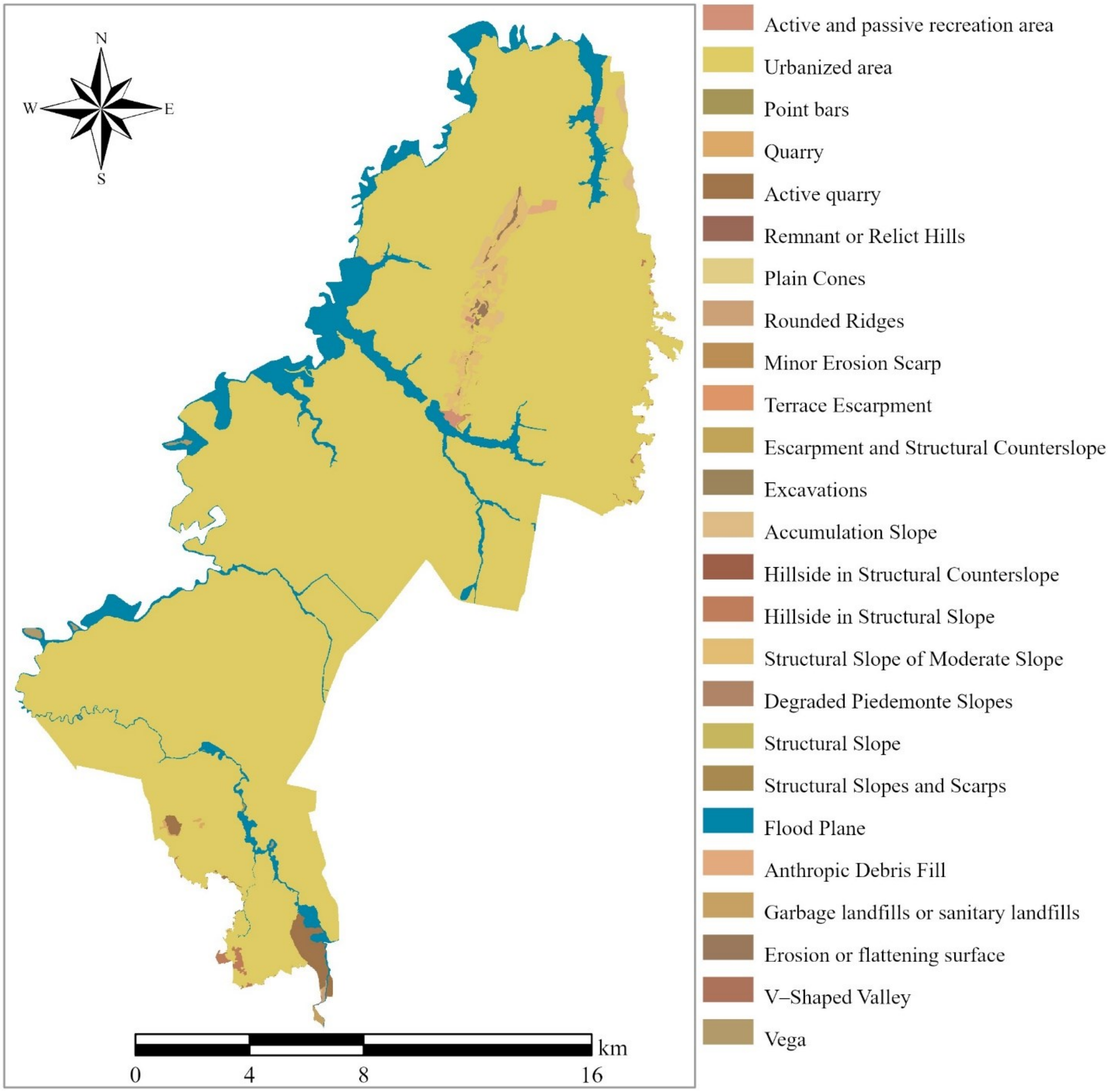


Figure S1. Urban geomorphology.

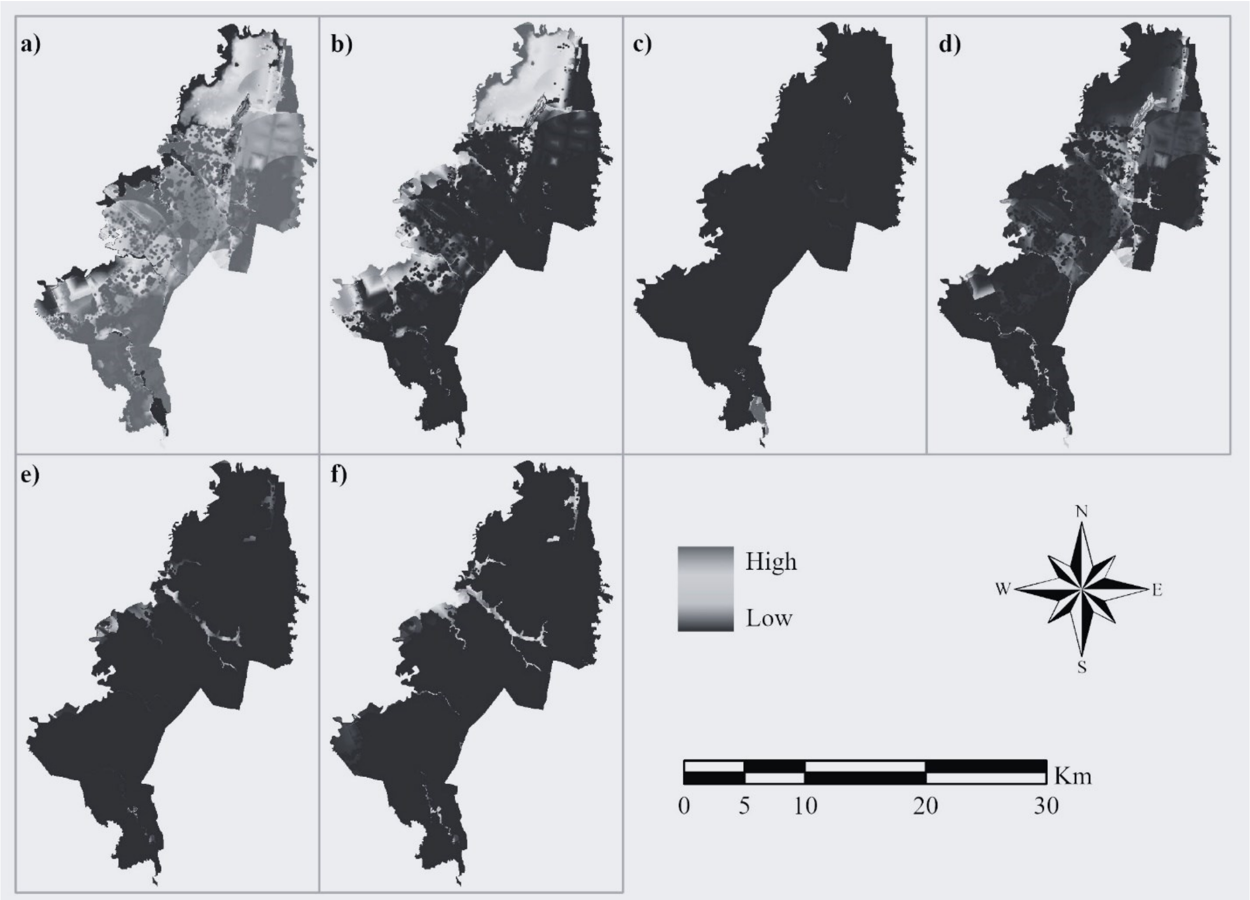


Figure S2. Land Use Land Cover transition probability maps for each land type – a) to constructions, b) to crops and pastures, c) to quarries, d) to urban green spaces, e) to water, f) to wetlands.

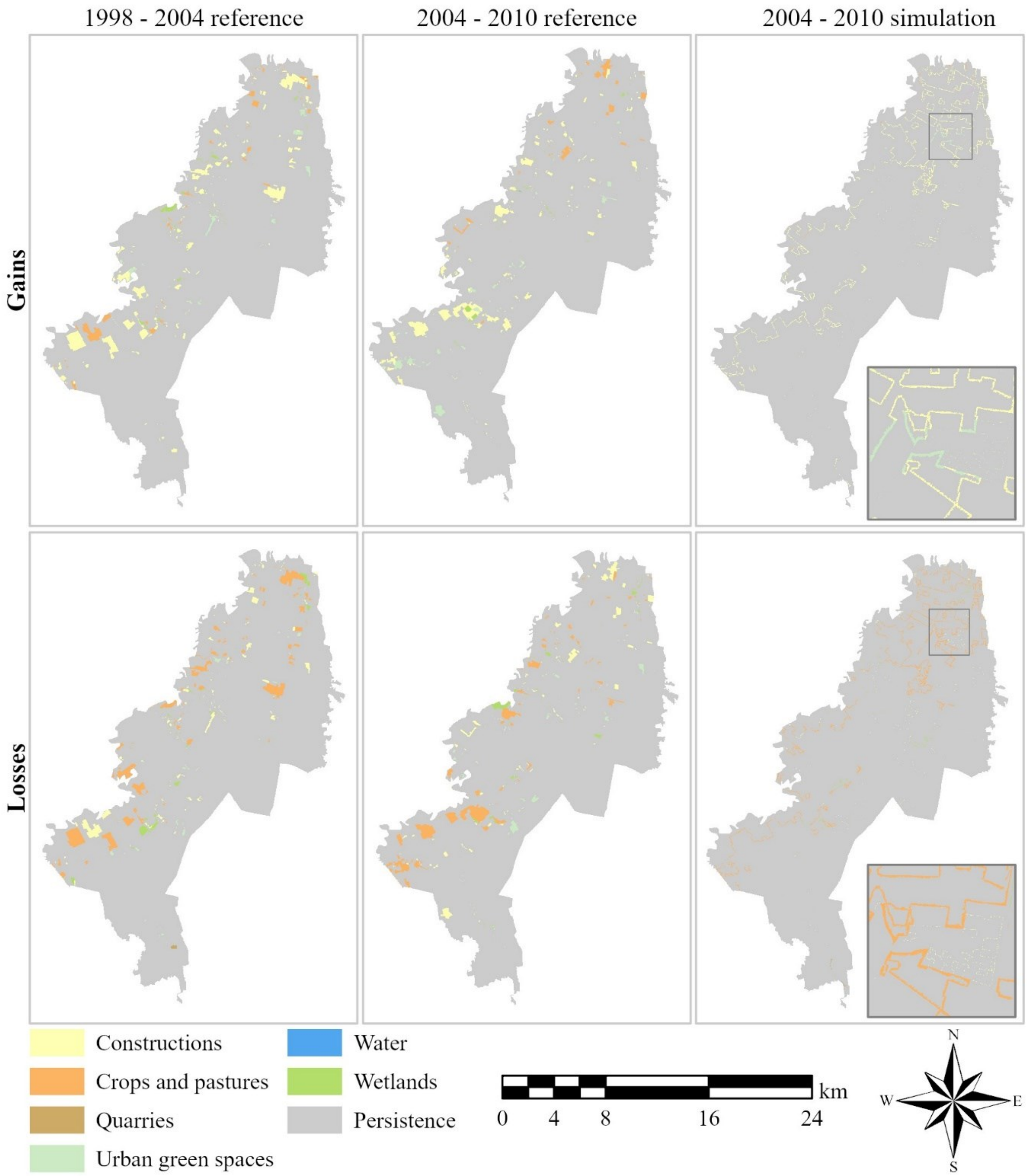


Figure S3. Change maps during the validation step.

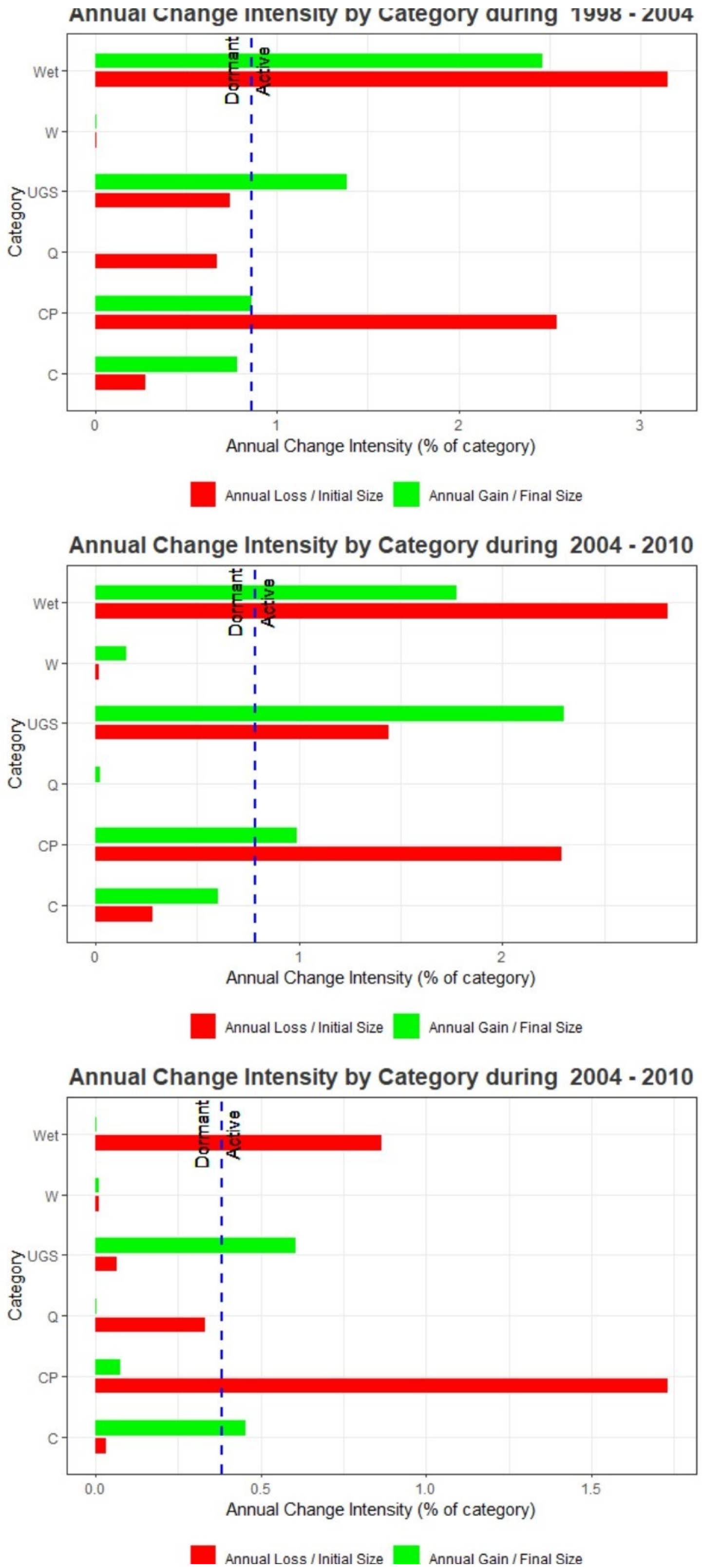


Figure S4. Category intensities during a) 1998-2004 reference, b) 2004-2010 reference, and c) 2004-2010 simulation. Wetlands (Wet), urban green spaces (UGS), crops and pastures (CP), quarries (Q), water (Wa), and constructions (C).

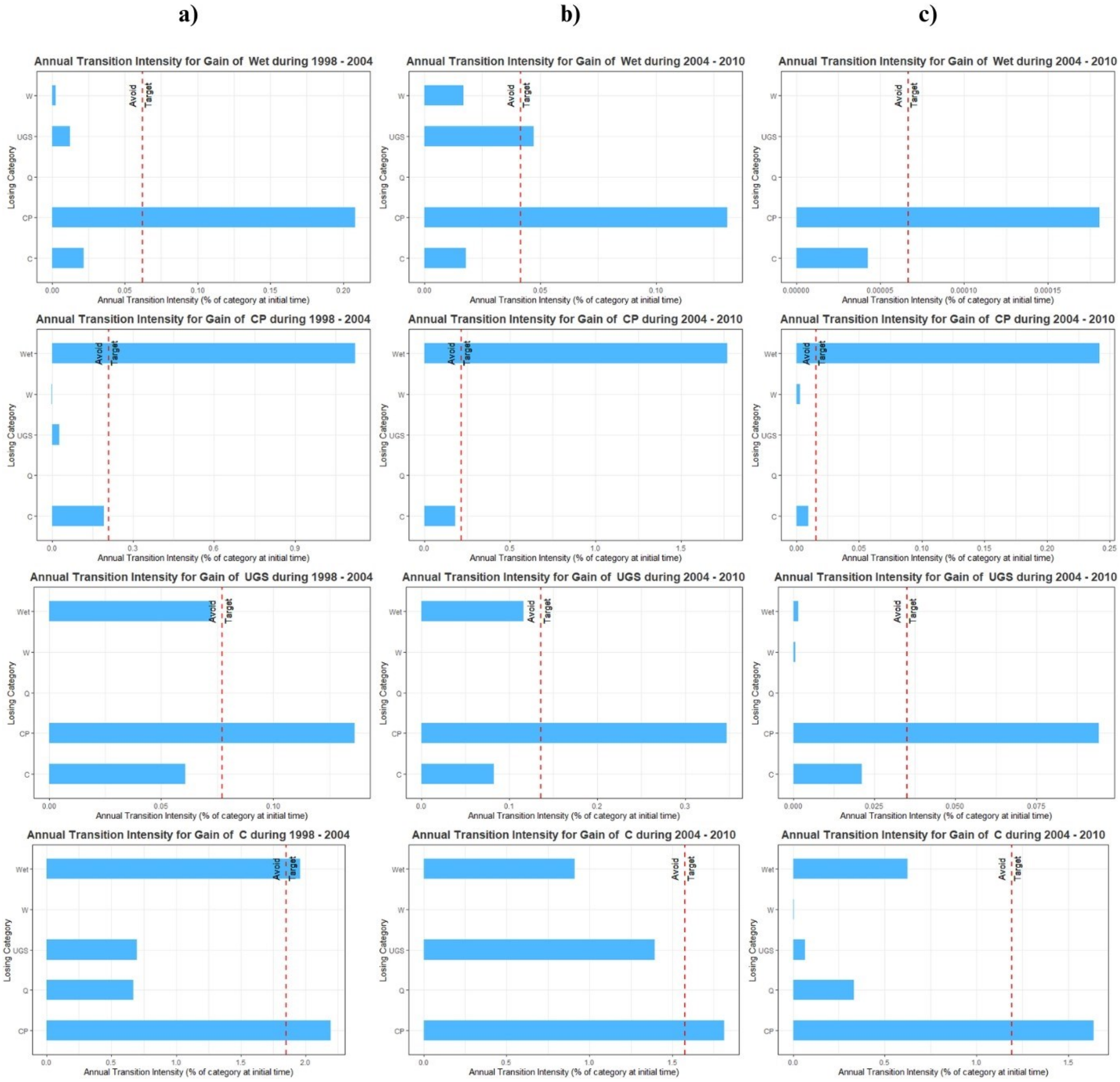


Figure S5. Transition intensity for the gains of wetlands (Wet), urban green spaces (UGS), crops and pastures (CP), water (W), quarries (Q), and constructions (C). Columns a) 1998-2004 reference, b) 2004-2010 reference, and c) 2004-2010 simulation.

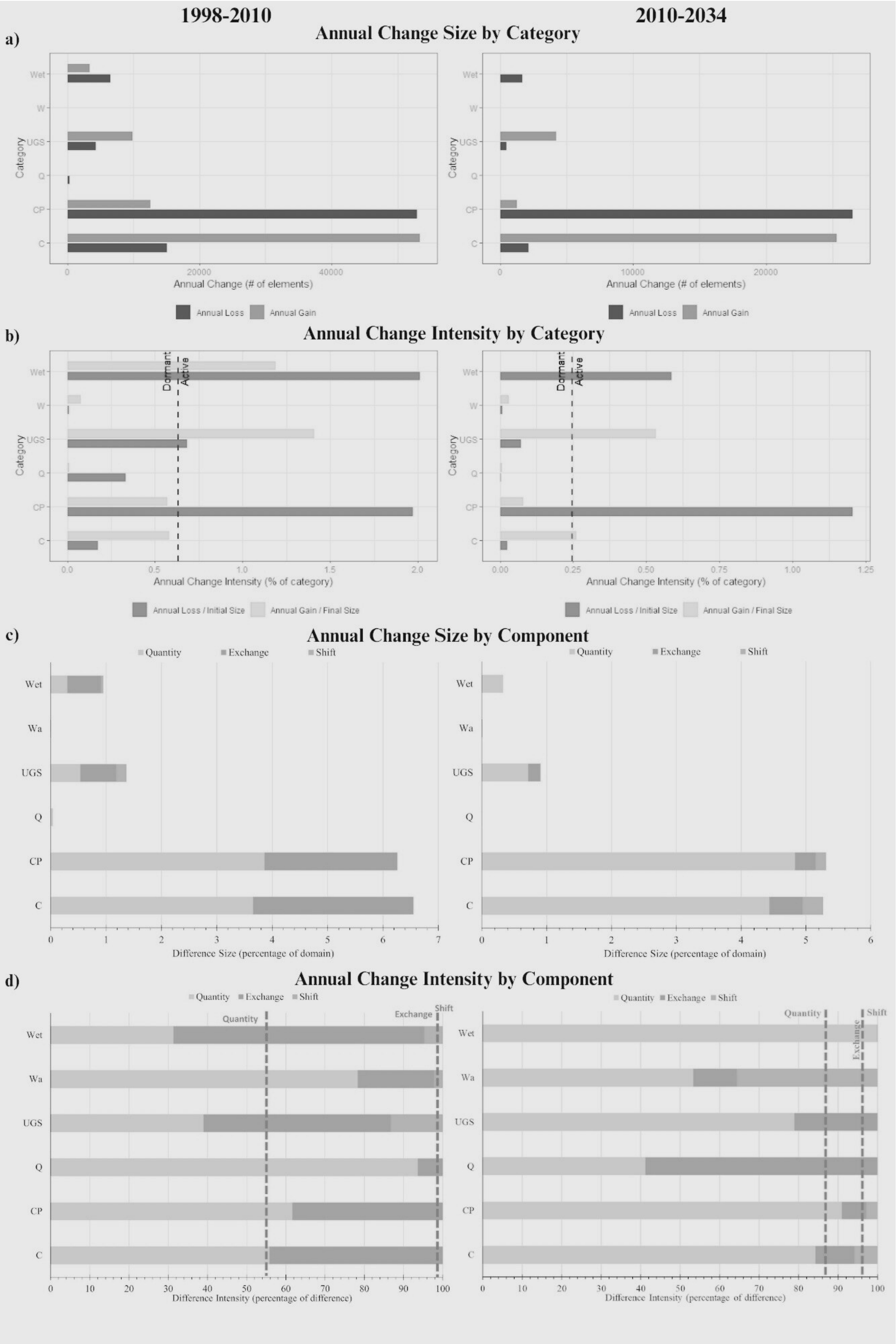


Figure S6. Black and white of Figure 7 in the manuscript.

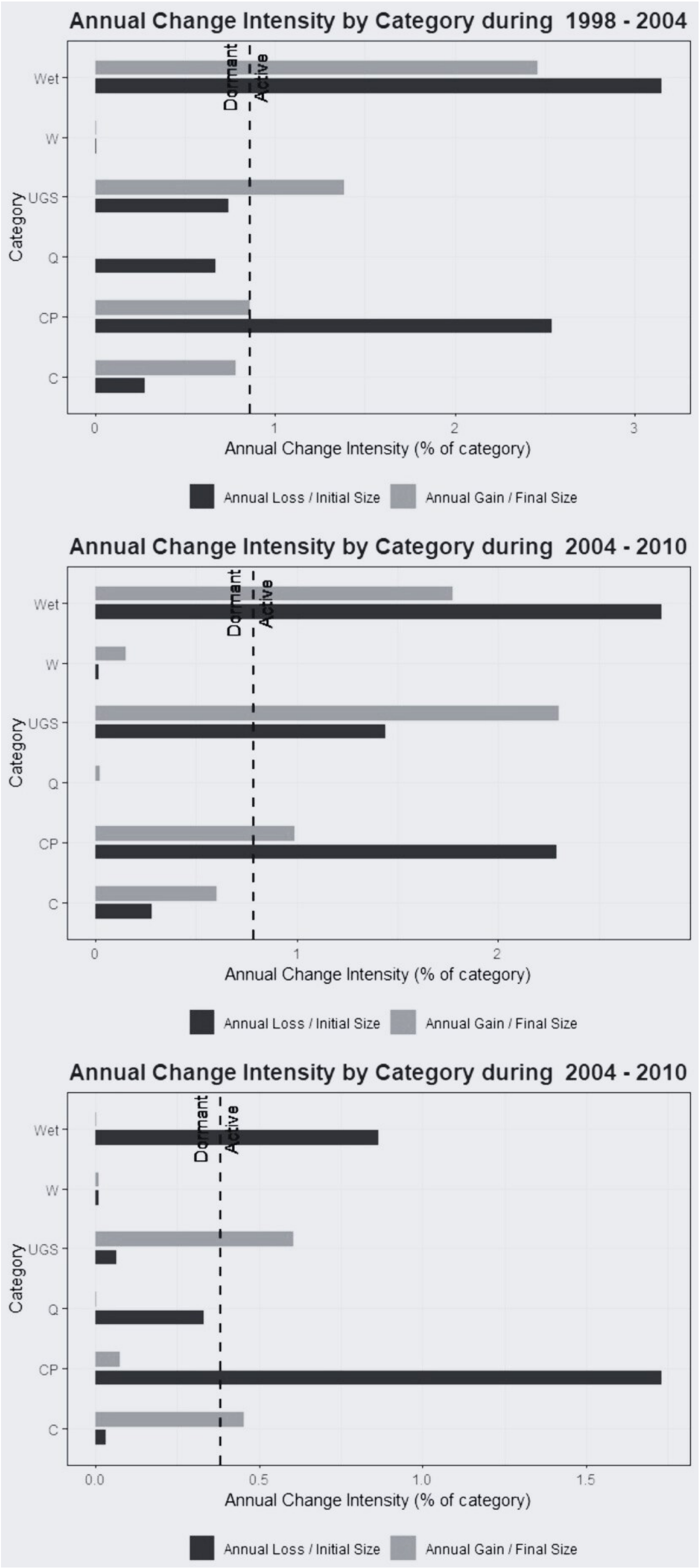


Figure S7. Black and white of Figure S4.
